# Group sequential designs in pragmatic trials: feasibility and assessment of utility using data from a number of recent surgical RCTs

**DOI:** 10.1186/s12874-022-01734-2

**Published:** 2022-10-01

**Authors:** Nick R. Parsons, Nigel Stallard, Helen Parsons, Aminul Haque, Martin Underwood, James Mason, Iftekhar Khan, Matthew L. Costa, Damian R. Griffin, James Griffin, David J. Beard, Jonathan A. Cook, Loretta Davies, Jemma Hudson, Andrew Metcalfe

**Affiliations:** 1 Statistics and Epidemiology Unit, Warwick Medical School, University of Warwick, CV4 7AL Coventry, UK; 2 Warwick Clinical Trials Unit (WCTU), Warwick Medical School, University of Warwick, CV4 7AL Coventry, UK; 3 University Hospitals Coventry and Warwickshire (UHCW), CV2 2DX Coventry, UK; 4 Nuffield Department of Orthopaedics, Rheumatology and Musculoskeletal Sciences (NDORMS), University of Oxford, OX3 7LD Oxford, UK; 5 Centre for Statistics in Medicine, Nuffield Department of Orthopaedics, Rheumatology and Musculoskeletal Sciences (NDORMS), University of Oxford, OX3 7LD Oxford, UK; 6 Health Services Research Unit (HSRU), University of Aberdeen, AB25 2ZD Aberdeen, UK

**Keywords:** Adaptive designs, Interim analysis, Early outcomes, Randomized controlled trials, Surgery

## Abstract

**Background:**

Assessing the long term effects of many surgical interventions tested in pragmatic RCTs may require extended periods of participant follow-up to assess effectiveness and use patient-reported outcomes that require large sample sizes. Consequently the RCTs are often perceived as being expensive and time-consuming, particularly if the results show the test intervention is not effective. Adaptive, and particularly group sequential, designs have great potential to improve the efficiency and cost of testing new and existing surgical interventions. As a means to assess the potential utility of group sequential designs, we re-analyse data from a number of recent high-profile RCTs and assess whether using such a design would have caused the trial to stop early.

**Methods:**

Many pragmatic RCTs monitor participants at a number of occasions (e.g. at 6, 12 and 24 months after surgery) during follow-up as a means to assess recovery and also to keep participants engaged with the trial process. Conventionally one of the outcomes is selected as the primary (final) outcome, for clinical reasons, with others designated as either early or late outcomes. In such settings, novel group sequential designs that use data from not only the final outcome but also from early outcomes at interim analyses can be used to inform stopping decisions. We describe data from seven recent surgical RCTs (WAT, DRAFFT, WOLLF, FASHION, CSAW, FIXDT, TOPKAT), and outline possible group sequential designs that could plausibly have been proposed at the design stage. We then simulate how these group sequential designs could have proceeded, by using the observed data and dates to replicate how information could have accumulated and decisions been made for each RCT.

**Results:**

The results of the simulated group sequential designs showed that for two of the RCTs it was highly likely that they would have stopped for futility at interim analyses, potentially saving considerable time (15 and 23 months) and costs and avoiding patients being exposed to interventions that were either ineffective or no better than standard care. We discuss the characteristics of RCTs that are important in order to use the methodology we describe, particularly the value of early outcomes and the *window of opportunity* when early stopping decisions can be made and how it is related to the length of recruitment period and follow-up.

**Conclusions:**

The results for five of the RCTs tested showed that group sequential designs using early outcome data would have been feasible and likely to provide designs that were at least as efficient, and possibly more efficient, than the original fixed sample size designs. In general, the amount of information provided by the early outcomes was surprisingly large, due to the strength of correlations with the primary outcome. This suggests that the methods described here are likely to provide benefits more generally across the range of surgical trials and more widely in other application areas where trial designs, outcomes and follow-up patterns are structured and behave similarly.

**Supplementary Information:**

The online version contains supplementary material available at 10.1186/s12874-022-01734-2.

## Background

Pragmatic clinical trials, that test interventions in everyday (routine practice) settings, typically have a number of important distinguishing characteristics that in large part determine their design and implementation [1, 2]. Primary amongst these are that they require large sample sizes (due to heterogeneity in the study population and interventions) and long follow-up periods in to order to assess effectiveness. One of the most important application areas for pragmatic trials is the assessment of surgical interventions [3, 4]; i.e. trials that involved a surgical intervention, or immediate postoperative intervention (e.g. wound management), in one or more arms of a study. Such interventions have historically been introduced based solely on what a surgeon believes might benefit patients; the perceived lack of rigour and inefficiency in surgical trials has motivated the development of many new processes and methodologies [5–7], and a consequent steady increase in the number of large randomised controlled trials (RCTs) over the last ten years. Many of the late-stage clinical trials testing surgical interventions are in Trauma and Orthopaedics (T&O). These trials are large, often because they use patient reported outcomes (PROMs) [2, 8], may take many (e.g. more than 5) years to complete due the long follow-up required and are consequently expensive. In order to improve both the efficiency and cost of testing new and existing surgical interventions, adaptive, and particularly group sequential, designs may have enormous potential and present an exciting opportunity for future research.

The 2022 START:REACTS clinical trial (Subacromial spacer for Tears Affecting Rotator cuff Tendons: a Randomised, Efficient, Adaptive Clinical Trial in Surgery)[9], stopped early and used a novel group sequential design, originally proposed by Parsons et al. [10] as a means to undertake clinical trials in a much more flexible and efficient manner, whilst retaining trial integrity. The approach proposed in the paper by Parsons et al. exploited the fact that it is very common in surgical trials to routinely monitor participants (often remotely) at a number of fixed occasions prior to collecting the definitive (final) study outcome (e.g. early outcomes might be collected at 3 and 6 months, prior to the main 12 month time-point). In such settings, if an interim analysis uses information from only those participants with final outcome data, then the opportunities for early stopping are likely to be limited simply by time constraints, as often trial recruitment will have completed prior to sufficient final outcome data being available for stopping decisions to be made. However, if the early outcomes are correlated with the final outcome, then a group sequential analysis [11] which uses the totality of information available from both early and final outcomes to estimate the treatment effect at the final study endpoint is likely to make adaptive designs feasible and lead to increases in statistical power [12–14].

Historically group sequential designs have not been used much, if at all, in surgical trials; a 2015 study [15] reported that only 1% of group sequential randomised controlled trials in peer-reviewed journals used a surgical intervention (60% used drugs, with the majority of RCTs in oncology). This, in part, reflects the fact that surgical trials have been behind other application areas in terms of the amount of rigorous research undertaken and the sophistication of the research methods employed. However, things have changed considerably in recent years with many more active research groups in the UK (where we are based) and around the world reporting the results of large (multicentre) RCTs in high-impact medical journals.

There are some universal barriers to the uptake of adaptive design methods that exist across all medical specialties, in particular the lack of knowledge, training and statistical expertise in research teams and more general anxiety about the impact of early stopping [16–18], which we will not address here. Provided we can overcome these more general barriers to uptake and specific concerns and issues around the appropriate methods to use and how they should be implemented, adaptive designs will likely become important and widely used methods in surgical RCTs. The group sequential design approach of Parsons et al. [10], was described in the context of a study (START:REACTS) comparing two treatment arms with two early outcome measures. The UK National Institute of Health Research (NIHR) funded study team (Efficacy and Mechanism Evaluation (EME) Programme, project reference 16.61.18) that undertook the START:REACTS study also investigated how the group sequential design methods used in the START:REACTS study might have been implemented and whether they would have resulted in changes in trial length and decision making in a number of recently undertaken high-profile conventional (fixed design) surgical trials in T&O. The main aims of this work were primarily to explore the generalisability of the methodological approach utilised in START:REACTS and assess whether this approach would have resulted in early stopping, using the original time sequence of patient recruitment data in these fixed design trials. This work is reported here using anonymized patient reported outcome data from seven T&O RCTs made available by Warwick Clinical Trials Unit (WCTU, Warwick Medical School; https://warwick.ac.uk/fac/sci/med/research/ctu/) and NDORMS, Nuffield Department of Orthopaedics, Rheumatology and Musculoskeletal Sciences (University of Oxford; https://www.ndorms.ox.ac.uk/).

A frequentist approach to the group sequential design is used here, defined by the error spent at each look with pre-defined information levels [11, 19, 20]. Bayesian methods are also widely available for adaptive group sequential designs [21], and have previously been suggested for trials in T&O and emergency medicine, albeit in very different applications to those presented here [22, 23]. Predominantly trials in T&O use patient reported outcome measures (PROMs), as the primary outcomes, which are typically assumed to be approximately normally distributed for the purposes of analysis. This is also the approach adopted here, as all the selected RCTs used PROMs; we do not discuss other (e.g. binary) outcome measures. However, we believe that the methods discussed could very easily be adapted for other outcomes and more generally, although the focus here is on surgery (and T&O), for trials in other application areas where settings (e.g. outcomes, patterns of follow-up and recruitment) are similar.

## Methods

### Data

Trial data from seven RCTs (see Table 1) were selected as typical of many recent surgical trials, in terms of the sample size, recruitment and participant follow-up periods and division of resources between primary and early outcome measures and for the pragmatic reason that the study principal investigators were able to respond quickly and positively to data sharing requests, and published protocols were available for all the studies. Unadjusted estimates of treatment effects and other key features are now described for each RCT; more detailed descriptions are available in an additional file [see Additional file 1]. We have chosen to use the data from trials unchanged, with the same sample sizes, for reasons of simplicity and ease of interpretation, rather than inflate the sample sizes as would be conventional to retain study power. In practice, we would typically increase the sample size, dependent on the number of planned interim analyses and stopping probabilities (see *Boundaries and information monitoring*), by a small amount to allow for the possible adaptations. Typically, this would make only a small or moderate change to the study sample size; for instance, in the 2022 START:REACTS clinical trial [9], the sample was increased from 170 to 188 (before allowance for missing data), to retain power at 90%.

**Table 1:** Brief details of selected RCTs

RCT	Sample size	Outcomes	Interventions (test,control)
WAT	$N=126$	OHS at 6w, 3m, 6m, 12m (primary)	RSA, THA
DRAFFT	$N=461$	PRWE at 3m, 6m, 12m (primary)	Plate, Wire
WOLLF	$N=460$	DRI at 3m, 6m, 9m, 12m (primary)	NPWT, Standard
FASHION	$N=348$	iHOT-33 6m, 12m (primary)	Surgery, PHT
CSAW	$N=210$	OSS at 6m (primary), 12m	ASAD, AMSR
FIXDT	$N=321$	DRI at 3m, 6m (primary), 12m	Plate, Nail
TOPKAT	$N=528$	OKS at 2m, 1y, 2y, 3y, 4y, 5y (primary)	PKR, TKR

#### WAT

The Warwick Arthroplasty Trial (WAT) was a two arm, parallel group, RCT conducted in the UK [24, 25], recruiting $N=126$ patients, between May 2007 and February 2010, suitable for a resurfacing arthroplasty of the hip. Patients were randomly assigned on a 1:1 basis to receive either a total hip arthroplasty (THA) or a resurfacing arthroplasty (RSA). The primary outcome was hip function, as measured by the patient-reported Oxford Hip Score (OHS; scale 0 to 48, with 48 representing no pain and perfect function) at 12 months (12m) after operation, with early outcome assessed at 6 weeks (6w), 3 months (3m) and 6 months (6m). The main result of the study was that there was no statistically significant difference in OHS between groups at 12 months; the mean score in the RSA group was 40.4 ($N1_{12m}=57$) and in the THA group 38.2 ($N0_{12m}=63$), a difference of 2.2 (95%CI; $-0.5$ to 12.6).

#### DRAFFT

The Distal Radius Acute Fracture Fixation Trial (DRAFFT) compared Kirschner wire fixation (Wire) with volar locking plate fixation (Plate) for $N=461$ patients with a dorsally displaced fracture of the distal radius recruited between July 2010 to July 2012 and randomised on a 1:1 basis [26, 27]. The trial used the Patient Rated Wrist Evaluation (PRWE; scale 0 to 100, with 100 being the worst score) score at 12 months (12m) after surgery to assess participants, with early assessments at 3 and 6 months. The main result of the study was that there was no statistically significant difference in PRWE score between groups at 12 months; the mean score in the Wire group was 15.3 ($N0_{12m}=211$) and in the Plate group 13.9 ($N1_{12m}=204$), a difference of 1.4 (95%CI; $-1.8$ to 4.5).

#### WOLLF

The Wound management of Open Lower Limb Fractures (WOLLF) trial was a multi-centre randomized trial performed in the UK Major Trauma Network, recruiting $N=460$ patients with a severe open fracture of the lower limb from July 2012 to December 2015 [28, 29]. Participants were randomized on a 1:1 basis to either negative pressure wound therapy (NPWT) or standard (Standard) wound management. The primary outcome of the study was the Disability Rating Index (DRI) score (range, 0 = no disability to 100 = completely disabled) at 12 months (12m), with early outcomes measured at 3, 6 and 9 months. The main result of the study was that there was no statistically significant difference in DRI score between groups at 12 months; the mean score in the NPWT group was 45.5 ($N1_{12m}=179$) and in the standard dressing group 42.4 ($N0_{12m}=195$), a difference of $-3.1$ (95%CI; $-8.5$ to 2.2).

#### FASHION

The Full UK RCT of Arthroscopic surgery for Hip Impingement versus best cONservative care trial (FASHION) was a pragmatic, multicentre, RCT recruiting $N=348$ adult patients, between July 2012 and July 2016, with femoroacetabular impingement syndrome, who were randomly allocated on a 1:1 basis to receive either hip arthroscopic surgery (Surgery) or personalised hip therapy (PHT) and followed-up at 6 and 12 months [30–32]. The primary outcome was the patient-reported International Hip Outcome Tool (iHOT-33; scale 0 to 100, with 100 representing no pain and perfect function) at 12 months after randomisation, with early outcome assessed at 6 months. The primary result of the study was that there was a statistically significant difference in iHOT-33 score between groups at 12 months; the mean score in the Surgery group was 58.8 ($N1_{12m}=158$) and in the PHT group 49.7 ($N0_{12m}=163$), a difference of 9.1 (95%CI; 3.3 to 14.9).

#### CSAW

The Can Shoulder Arthroscopy Work (CSAW) trial was a three arm trial, but we limit discussion here to the main treatment comparison. The CSAW RCT randomized $N=210$ participants (on a 1:1 basis), from September 2012 to June 2015, to either Arthroscopic SubAcromial Decompression (ASAD) or Active Monitoring with Specialist Reassessment (AMSR; no surgical treatment) and used the Oxford Shoulder Score (OSS; scale 0 to 48, with 0 being the worst score) at 6 months after randomisation to assess outcomes [33, 34]. OSS was also assessed at 12 months after randomisation, but no early assessment of OSS was made before the 6 months primary endpoint. The primary result of the study was that there was no statistically (or clinically) significant difference in OSS at 6 months between groups; the mean score in the ASAD group was 32.7 ($N1_{6m}=90$) and in the AMSR group 29.4 ($N0_{6m}=90$), a difference of 3.3 (95%CI; $-0.2$ to 6.8).

#### FIXDT

The FIXation of Distal Tibia fractures (FIXDT) trial recruited $N=321$ patients between April 2013 and April 2016 and compared intramedullary nail fixation (Nail) with locking plate fixation (Plate) for adult patients with a displaced fracture of the distal tibia [35, 36] using the Disability Rating Index (DRI; range 0 to 100, with 100 being completely disabled) at 6 months (6m), with early outcome measured at 3 months (3m) and long-term outcome assessed at 12 months. The primary result of the study was that there was no statistically significant difference in DRI score between groups at 6 months; the mean score in the Nail group was 29.8 ($N1_{6m}=142$) and in the Plate group 33.8 ($N0_{6m}=140$), a difference of $-4.0$ (95%CI; $-9.6$ to 1.6).

#### TOPKAT

The Total Or Partial Knee Arthroplasty Trial (TOPKAT) randomized $N=528$ participants (on a 1:1 basis) from January 2010 to September 2013 and compared total knee replacement (TKR) to partial knee replacement (PKR) for patients with medial compartment osteoarthritis of the knee using the Oxford Knee Score (OKS; scale 0 to 48, with 0 being the worst score) at 5 years (5y) after randomisation with early outcomes assessed at 2 months (2m) and on a yearly basis at 1, 2, 3 and 4 years [37, 38]. The primary result of the study was that there was no statistically significant difference in OKS between groups at 5 years; the mean score in the TKR group was 37.0 ($N0_{5y}=231$) and in the PKR group 38.0 ($N1_{5y}=233$), a difference of 1.0 (95%CI; $-0.4$ to 2.5).

### Adaptive group sequential designs

#### Overview

This study assesses whether the RCTs described here, which were originally implemented using conventional fixed sample size designs, would have stopped early if an adaptive (group sequential) trial design had been used. For the purposes of this work, all the selected RCTs had two treatment arms (with one nominally designated as the control or standard treatment), randomized participants to treatment groups in a 1:1 ratio and reported a single primary outcome, with one or more assessments of the trial outcome measure (e.g. outcomes at 3 and 6 months or 1, 2, 3, 4 and 5 years). In order to assess whether the trial would have stopped early, the temporal sequence of data accumulation was replicated in *exactly* the manner it was in the original trial using the dates (which were available from the original trial databases) when each outcome measure was made. Using the original trial data, and selected options for the number of planned interim analyses and stopping boundaries, we will simulate how each study might have progressed using the methodological approach described by Parsons et al. for an adaptive two-arm clinical trial using early endpoints to inform decision making; this methodology is described in detail in an additional file [see Additional file 2]. The approach employed here, using available data from recent trials to retrospectively assess the utility of alternative (adaptive) designs, is similar in spirit to a number of others studies; see for instance [39] (Chapter 7).

To simulate a single instance of an adaptive trial the following procedure was implemented: (i) we decided on the number of interim analyses we wished to make, stopping probabilities and the information levels necessary to trigger the interim analyses; (ii) these settings were used to determine upper and lower stopping boundaries for the test statistics using pre-specified alpha-spending functions; (iii) data from the original trial were used to simulate information accrual in the new adaptive design, using the observed ordering of data accumulation from the original trial; (iv) when an information threshold was hit, a test statistic was calculated using all the available information, from the final (primary) and all early endpoints; (v) the test statistic was compared to the boundaries, with decisions on stopping following from this process; (vi) if the decision was to continue, more information was accrued and any additional interim analyses implemented until the final planned interim analysis.

#### Treatment effect estimates

The primary interest of all the RCTs discussed here was to estimate the effect of the test treatment, on the study outcome at the definitive (final) endpoint, time *t* (the primary study endpoint), which we hereafter call $\beta _{t}$. In the simplest possible case a primary outcome is measured at time *t* only and these data alone inform the estimate of the treatment effect $\beta _{t}$. However, if early outcomes (at times before *t*) are available, then they can provide information on the final outcome due to the correlation between the early and the final outcomes for each participant. A strong correlation ($\rho$) between, for instance, 3 and 6 month outcomes suggests that a good (or poor) outcome at 3 months will be indicative of a good (or poor) outcome at 6 months. Therefore, fitting longitudinal models to the time course of data allows one to exploit this early information to improve estimates of the treatment effects, through improved precision in estimating $\beta _{t}$; this strategy for decision making in the setting of an adaptive design as been discussed previously [10, 12–14]. To be clear, in this model, treatment effects for the early outcomes *per se* do not provide information on treatment effects for the final trial outcome $\beta _{t}$. The notation used here for the effect size estimate ($\beta _{t}$) reflects the fact that estimation follows from fitting a longitudinal linear model to the totality of outcome data. Methods for estimating $\beta _{t}$ and $\text {var}(\beta _{t})$ and example code in R, using all the data available at any time-point during follow-up (FU), are provided in an additional file [see Additional file 2]. The test statistic $Z =\beta _{t}/\mathrm {sd}(\beta _{t})$ is used to make stopping decisions at the interim analyses using estimates of the covariance parameters (i.e. the correlations between outcomes $\rho$ and standard deviations of the outcomes $\sigma$). The interim analyses are triggered at pre-set (expected) information thresholds, with observed information during recruitment and follow-up given by $I=1/\text {var}(\beta _{t})$. In addition, as a means to assess the importance of the early outcome data in modifying estimates of the $Z = \beta _{t}/\mathrm {sd}(\beta _{t})$, an analysis was undertaken that forced all the correlations to be zero; i.e. an analysis that uses final outcome data only. We designate these parameters, which show the evidence for treatment effects using final outcome data only, as $\beta 0_{t}$ and $\text {var}(\beta 0_{t})$, with $Z0=\beta 0_{t}/\mathrm {sd}(\beta 0_{t})$ and $I0=1/\text {var}(\beta 0_{t})$.

Estimates $\beta _{t}$ can be obtained at each of the analyses. At the final analysis at the end of the trial, if complete follow-up data are available for all participants, then $\beta 0_{t}$ and $\text {var}(\beta 0_{t})$ will be equal to $\beta _{t}$ and $\text {var}(\beta _{t})$. However, for all the RCTs in this study there are some missing data such that there are a number of participants who did not provide final outcomes but had one or more early outcomes. If these early outcomes are correlated with the final outcomes, then they will provide some information on the final outcomes and cause estimates of treatment effects $\beta _{t}$ to be somewhat different from $\beta 0_{t}$, and also cause the former to have smaller variances than the latter. If we were reporting a conventional prospectively planned and implemented group sequential trial, rather than the *simulated* retrospective trials reported here, then we would generally need to adjust effect estimates for potential bias due to the interim analyses; for instance using Todd’s approach [40]. However, here we focus purely on the unadjusted effect estimates and stopping decisions, mainly for simplicity of exposition, as it precludes the need to make adjustments for every different setting of the boundaries for each trial.

#### Interim analyses

The number of feasible interim analyses for each RCT were determined, in large part, by the *expected* patterns of recruitment and data accumulation for each RCT. Interim analyses need to occur during the *window of opportunity* bounded at the start by the earliest time sufficient data are available for a sensible analysis to occur and at the end by the time when recruitment is completed. After the latter time-point, there is no advantage to stopping a study, as conventionally all participants recruited into the trial should complete follow-up. The number of possible interim analyses for each RCT was determined, before simulating data accumulation for the adaptive design, by a consideration of the likely width of the window of opportunity, which is itself determined by the likely pattern of recruitment and follow-up. We have endeavoured, where possible, to use only the information that would have been available to those designing the trials at the initial stages when decisions about the likely number of analyses would need to have had to be made. The lead statisticians from all of the selected trials were consulted on these issues, and the knowledge gained from them and from the published protocols for all the trials was used to inform the designs for each RCT. Details of the original sample fixed design size calculation for each RCT can be found in an additional file [see Additional file 2]. Clearly, if the selected RCTs had been prospectively planned as adaptive designs, then some adjustment to the sample size would have been made to maintain power at the required level. We make no attempt to increase the sample size, to maintain power, in this study but rather focus solely on the stopping decisions at the interim analyses.

#### Boundaries and information monitoring

Given the practical constraints imposed by the need for interim analyses to take place during the window of opportunity, we restrict this study to a maximum of three interim analyses, in addition to the final analysis, within any trial. The primary focus of this study is to assess whether and under what circumstances a group sequential design may have resulted in the selected trials stopping early. Many complex interventions (e.g. surgery or physiotherapy) tested in pragmatic publicly funded trials, unlike in the pharmaceutical industry, are licensed for use, without a requirement for information on efficacy that would be required to get them used in practice [41]. Adaptive designs methods, that are regularly applied in industry, have for the most part not been used in publicly funded trials [42], and this fact in large part provided the motivation for the selection of the trials described here. They are all publicly funded trials of complex interventions, typically incorporating health economic analysis, in difficult settings, with logistical and practical issues that many believe make adaptive trials difficult or almost impossible. We do not share this view, but rather believe that study designs using early looks at emerging data to assess stopping would have been perfectly possible and good options for all the selected trials. An early futility assessment has the potential to increase efficiency, save patients and decrease costs in publicly funded trials, and many trialists and statisticians suggest that, where possible, investigators should aim to include a futility analysis in their designs for such trials [41]. For these reasons, and the possibility of obtaining more enlightening results, we choose to focus mainly on futility stopping in our work. If we had chosen trials of a very different type (i.e. testing simple interventions), then we would likely have placed a much greater focus on efficacy stopping. We choose to adopt a range of previously suggested futility boundaries [10]; which we label as (a-d). These are defined by stopping probabilities in the setting of up to three interim analyses, that represented a sequence of four increasingly aggressive options, from a low probability of stopping for futility, labelled as (a), to a high probability, labelled as (d), with (b) and (c) intermediate to these. Table 2 shows the probabilities of stopping and rejecting the null hypothesis (H0) in favour of alternative ($\alpha ^{*}_{u}$; efficacy), and the probabilities of stopping without rejecting H0 ($\alpha ^{*}_{l}$; futility), for the four settings (a-d) for one, two and three interim analyses, under the null hypothesis that there is no difference between the two treatment groups. The stopping probabilities from Table 2 are used to construct appropriate boundaries for standardized test statistics at each of the planned interim analyses for each trial. This required us to make some assumptions, based on what we believe the trial team may have thought prior to the commencement of recruitment, about (i) the number of possible interim analyses, (ii) the expected standard deviations ($\sigma ^{*}_{t}$) and correlations ($\rho ^{*}_{s,t}$) between the early and final endpoints and (iii) the number of data-points that may have been available at each of the interim analyses; we use the $*$ notation to distinguish expected values from observed values hereafter. Values for $\sigma ^{*}$ were taken from the original (fixed design) sample size calculations, reported in the published trial protocols. Whereas $\rho ^{*}_{s,t}$, which were generally unknown, were arbitrarily set for all pairs of outcomes to be $\rho ^{*}_{s,s^{\prime }}=0.5$, to reflect an expectation of moderate to strong associations. In reality if the trials had been planned prospectively using a group sequential design, more realistic estimates of $\rho ^{*}_{s,t}$ would have been used (e.g. from historical or pilot data) to determine stopping boundaries. The expected values of the covariance parameters are used to calculate the expected information necessary to trigger each interim analysis ($I^{*}$), which alone, together with the settings of Table 2, allow us to define stopping boundaries for the observed test statistics for the settings (a-d) for up to three interim analyses; further details can be found in an additional file [see Additional file 2].

**Table 2:** Four test settings (a-d) for futility and efficacy stopping with cumulative probabilities under the null hypothesis, $\alpha ^{*}_{l}$ and $\alpha ^{*}_{u}$ for one, two and three interim analyses

Interims	$\alpha ^{*}_{l}$				$\alpha ^{*}_{u}$
	(a)	(b)	(c)	(d)	
One interim analyses					
1	0.160	0.320	0.480	0.640	0.005
End	0.975	0.975	0.975	0.975	0.025
Two interim analyses					
1	0.080	0.160	0.240	0.320	0.001
2	0.160	0.320	0.480	0.640	0.010
End	0.975	0.975	0.975	0.975	0.025
Three interim analysis					
1	0.080	0.160	0.240	0.320	0.001
2	0.160	0.320	0.480	0.640	0.005
3	0.240	0.480	0.720	0.960	0.010
End	0.975	0.975	0.975	0.975	0.025

#### Implementation

For each of the selected RCTs, data were simulated using the observed recruitment, such that they represent the order that data would have accumulated in real time (i.e. in the order data would have accumulated in the original trial). Information monitoring begins, after sufficient data are available to estimate accumulated information, and continues on a regular basis (every two weeks) to reflect what would likely have happened in the trial, if an adaptive design had been implemented. Once the required information level, to trigger an interim analysis, is reached, the test statistic is calculated and compared to the stopping boundaries. Decisions about whether the *simulated* group-sequential trial would have stopped, either for efficacy or futility, are made by comparing the estimated test statistics at each interim analysis to the stopping boundaries for the four scenarios (a) to (d). As a comparison, for trials that are stopped, at the interim analysis data on all those study participants recruited up to that point were used to estimate model parameters in an overrunning analysis [43, 44]; this analysis comprised all the data (complete follow-up) that would eventually have accumulated on those participants already recruited. This process of data collection and decision making is continued for subsequent interim analyses or until data accumulation is complete. This simulated process of data monitoring and analysis is exactly equivalent to how the process proceeded in the recently reported START:REACTS study [9], which proved to work well and efficiently for the study statistician (who oversaw the routine information monitoring) and the trial team.

## Results

**Fig. 1:**
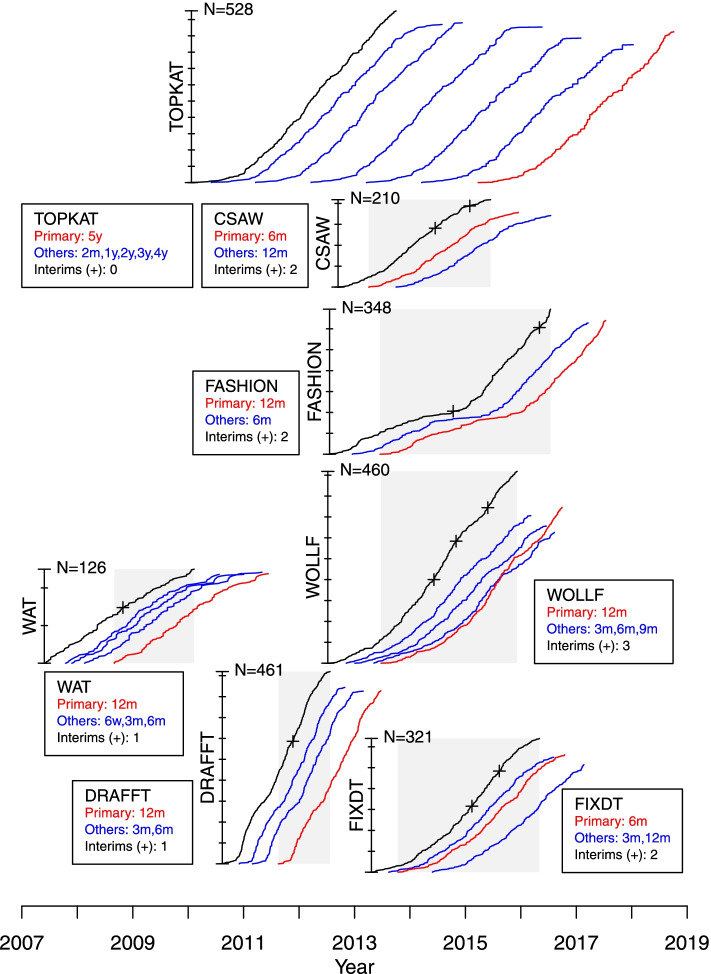
Progress of recruitment and follow-up for each RCT. The schematic shows recruitment accrual curves, total recruitment, colour coded follow-up accrual curves for the primary and all endpoints, the window of opportunity for stopping (shaded) and planned occasions for the interim analyses for each RCT, which are located in temporal sequence horizontally for the period 2007 to 2019. The vertical placement of each RCT is for representational purposes only and does not signify any characteristic of importance to the conduct of the trial

**Fig. 2:**
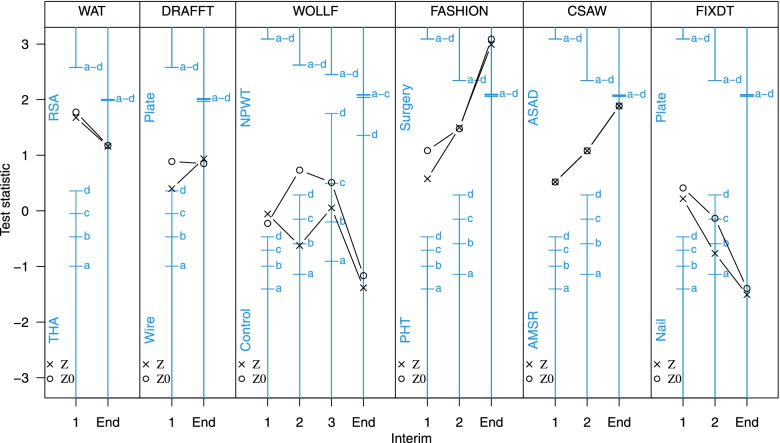
Stopping boundaries and test statistics for each RCT. Stopping boundaries are shown for each of the four selected setting (a-d), together with test statistics (*Z* and *Z*0) at each interim analysis and trial end for each RCT

The recruitment accrual curves, windows of opportunity for stopping (shaded) and planned numbers and occasions for the interim analyses are shown schematically for each the *simulated* or *re-imagined* group-sequential trials in Fig. 1. A detailed description of the results of each of the *simulated* group-sequential trials is provided in an additional file [see Additional file 1]; for each of the seven selected RCTs this file shows the calculation and justification for the upper and lower stopping boundaries, the numbers of participants providing early and final outcome data, treatment group means and estimates of treatment effects, test statistics, correlations and variances at each interim analysis at overrunning. The progress of the *simulated* group-sequential trials is summarised in Fig. 2 which shows stopping boundaries, for all settings (a-d), and test statistics for each RCT, indicating where boundaries where crossed. The most important of the results from model fitting are also presented here in Tables 3, 4 and 5; these show estimates of the treatment effects and test statistics, correlations and standard deviations and numbers of participants and progress (in months) for each interim analysis for each trial respectively. The results are summarised in the following for each trial in turn.

**Table 3:** Estimates of the treatment effects ($\beta _{t}$ and $\beta 0_{t}$) on the primary outcome at time *t*, test statistics (*Z* and *Z*0) and information accrual (*I*), at each interim analysis and the study end, for each RCT; where $Z=\beta _{t}/\mathrm {sd}(\beta _{t})$, $Z0=\beta 0_{t}/\mathrm {sd}(\beta 0_{t})$ and $I=1/\text {var}(\beta _{t})$ and $I0=1/\text {var}(\beta 0_{t})$. The primary outcome time-point *t* and the expected information $I^{*}$, to trigger each interim analysis, are shown for each RCT

RCT	Early and primary			Primary only		
Interim	$\beta _{t}$	*Z*	*I*	$\beta 0_{t}$	*Z*0	*I*0
WAT ($t=12m$ and $I^{*}=$ 0.150 and 0.321)						
1	4.30	1.68	0.152	6.80	1.78	0.068
End	2.18	1.16	0.283	2.23	1.18	0.278
DRAFFT ($t=12m$ and $I^{*}=$ 0.073 and 0.219)						
1	1.41	0.40	0.080	4.39	0.89	0.041
End	1.51	0.94	0.387	1.37	0.85	0.384
WOLLF ($t=12m$ and $I^{*}=$ 0.025, 0.050, 0.075 and 0.165)						
1	-0.36	-0.06	0.025	-1.79	-0.22	0.016
2	-2.76	-0.62	0.051	4.11	0.73	0.032
3	0.19	0.05	0.075	2.16	0.51	0.056
End	-3.65	-1.38	0.143	-3.14	-1.16	0.137
FASHION ($t=12m$ and $I^{*}=$ 0.025, 0.050 and 0.127)						
1	3.60	0.58	0.026	7.08	1.08	0.023
2	6.50	1.50	0.053	6.77	1.48	0.048
End	8.74	2.99	0.117	9.08	3.09	0.116
CSAW ($t=6m$ and $I^{*}=$ 0.123, 0.247 and 0.525)						
1	1.42	0.52	0.134	1.42	0.52	0.134
2	2.17	1.08	0.249	2.17	1.08	0.249
End	3.31	1.89	0.325	3.31	1.89	0.325
FIXDT ($t=6m$ and $I^{*}=$ 0.036, 0.071 and 0.165)						
1	1.14	0.22	0.037	2.29	0.41	0.033
2	-2.85	-0.76	0.072	-0.52	-0.13	0.066
End	-4.27	-1.51	0.125	-3.97	-1.39	0.124

**Table 4:** Estimates of correlations between early and primary outcomes ($\rho _{s,t}$) and standard deviations ($\sigma$), at each interim analysis and the study end, for each RCT; the expected correlations were $\rho ^{*}_{s,s^{\prime }}=0.5$ for all pairs of outcomes for all RCTs and the primary outcome time-point *t* and expected standard deviation ($\sigma ^{*}_{t}$) are shown for each RCT

RCT	Correlations ($\rho$)			Standard deviations ($\sigma$)			
Interim	$\rho _{1,t}$	$\rho _{2,t}$	$\rho _{3,t}$	$\sigma _{1}$	$\sigma _{2}$	$\sigma _{3}$	$\sigma _{t}$
WAT (FU at 6*w*, 3*m*, 6*m* and $t=12m$ and $\sigma ^{*}_{t}=9$)							
1	0.60	0.71	0.50	9.9	10.4	9.7	5.6
End	0.59	0.72	0.80	10.1	9.8	8.9	10.4
DRAFFT (FU at 3*m*, 6*m* and $t=12m$ and $\sigma ^{*}_{t}=20$)							
1	0.78	0.72	-	22.3	17.5	-	13.5
End	0.61	0.73	-	22.6	18.2	-	16.6
WOLLF (FU at 3*m*, 6*m*, 9*m* and $t=12m$ and $\sigma ^{*}_{t}=25$)							
1	0.59	0.77	0.88	22.6	23.5	26.7	25.7
2	0.58	0.78	0.89	22.1	23.9	24.7	24.7
3	0.48	0.71	0.82	21.1	23.6	25.2	26.4
End	0.58	0.73	0.74	21.3	23.9	25.6	26.2
FASHION (FU at 6*m* and $t=12m$ and $\sigma ^{*}_{t}=20$)							
1	0.56	-	-	22.9	-	-	25.8
2	0.56	-	-	23.9	-	-	27.3
End	0.57	-	-	24.1	-	-	26.3
CSAW (FU at $t=6m$ and $\sigma ^{*}_{t}=9$)							
1	-	-	-	-	-	-	12.2
2	-	-	-	-	-	-	11.7
End	-	-	-	-	-	-	11.8
FIXDT (FU at 3*m* and $t=6m$ and $\sigma ^{*}_{t}=20$)							
1	0.61	-	-	20.1	-	-	24.6
2	0.65	-	-	20.1	-	-	23.7
End	0.65	-	-	20.0	-	-	24.1

**Table 5:** Numbers of participants (*N*) and progress in trial recruitment (total numbers of participants and months of recruitment), at each interim analysis and the study end, for each RCT; the primary outcome time-point *t* and follow-up (FU) time-points are shown for each RCT

RCT	Numbers (*N*)				Progress	
Interim	$N_{1}$	$N_{2}$	$N_{3}$	$N_{t}$	Total	Months
WAT (FU at 6*w*, 3*m*, 6*m* and $t=12m$)						
1	49	43	29	10	75	17
End	119	119	122	120	126	48
DRAFFT (FU at 3*m*, 6*m* and $t=12m$)						
1	205	135	-	26	294	15
End	423	414	-	415	461	34
WOLLF (FU at 3*m*, 6*m*, 9*m* and $t=12m$)						
1	115	84	51	37	201	23
2	188	136	85	74	293	27
3	255	217	173	156	373	34
End	354	329	314	374	460	50
FASHION (FU at 6*m* and $t=12m$)						
1	86	-	-	62	104	27
2	208	-	-	141	304	45
End	315	-	-	321	348	59
CSAW (FU at $t=6m$)						
1	-	-	-	79	142	21
2	-	-	-	137	195	28
End	-	-	-	180	210	39
FIXDT (FU at 3*m* and $t=6m$)						
1	105	-	-	79	159	21
2	178	-	-	146	243	27
End	273	-	-	282	321	42

### WAT

A single interim analysis was planned for the *simulated* group-sequential WAT trial. For all four boundary settings tested, the WAT study would not have stopped at the interim analysis, when data were available from $N_{12m}=10$ participants with 12m outcomes, $N_{6m}=29$ with 6m outcomes, $N_{3m}=43$ with 3m outcomes and $N_{6w}=49$ with 6w outcomes. At this interim analysis $N=75$ participants had been recruited into the study and follow-up would have been completed in 17 months; this compares to $N=126$ and 48 months for the original study. The expected standard deviation of the primary outcome ($\sigma ^{*}_{12m}=9$), used in the original sample size calculation, and used to build the group-sequential design, was much larger than the observed value at the interim analysis ($\sigma _{12m}=5.6$). This caused the interim analysis to take place at a much earlier time than planned (i.e. with fewer participants with 12m outcomes than expected; $N_{12m}=10$ rather than the expected $N^{*}_{12m}=40$). However, given the small, but not clinically significant, result observed in the original study, it seems unlikely that any sensible stopping rule would have caused the WAT study to stop early.

### DRAFFT

A single interim analysis was planned for the *simulated* group-sequential DRAFFT trial. For all four boundary settings tested, the DRAFFT study would not have stopped at the interim analysis, when data where available from $N_{12m}=26$ participants with 12m outcomes, and $N_{6m}=135$ with 6m outcomes and $N_{3m}=205$ with 3m outcomes. At this interim analysis $N=294$ participants had been recruited into the study and follow-up would have been completed in 15 months; this compares to $N=461$ and 34 months for the original study. The expected standard deviation of the primary outcome ($\sigma ^{*}_{12m}=20$), used in the original sample size calculation, and used to build the group-sequential design, was larger than the observed value ($\sigma_{12m}=13.5$). This, together with the larger than expected correlations ($\rho _{3m,12m}=0.78$ and $\rho _{6m,12m}=0.72$; c.f. $\rho ^{*}_{s,t}=0.5$), caused the interim analysis to take place at a much earlier time than planned (i.e. with fewer participants with 12m outcomes than expected; $N_{12m}=26$ rather than the planned $N^{*}_{12m}=100$). The original DRAFFT study recruited at a much faster rate than expected, and the sample size was increased from $N=390$ to $N=461$. This gave greater precision in the estimate of the treatment effect, which was important for inferences and the health economics analysis particularly [45].

### WOLLF

Three interim analyses were planned for the *simulated* group-sequential WOLLF trial. For three of the four boundary settings tested (a-d), the WOLLF study would have stopped for futility, a result consistent with the original study, at the second interim analysis, when data were available from $N_{12m}=74$ participants with 12m outcomes, $N_{9m}=85$ with 9m, $N_{6m}=136$ with 6m and $N_{3m}=188$ with 3m outcomes. At this interim analysis $N=293$ participants had been recruited into the study and follow-up would have been completed in 27 months; this compares to $N=460$ and 50 months for the original study. The estimated treatment effect ($\beta _{12m}$) for the 12m DRI outcome at the second interim analysis was -2.8 (95%CI; -11.4 to 5.9) favouring the control treatment (and -3.5 for the overrunning analysis); the estimate of the treatment effect at the trial end in the original (fixed design) study was -3.1 (95%CI; -8.5 to 2.2). At the second interim analysis when the study would have stopped, the estimated treatment effect from the model was -2.8 (favouring the control), whereas the raw difference between groups using 12m data only ($\beta 0_{12m}$) was 4.1 (favouring NPWT). There were extremely strong correlations between the early outcomes (3m, 6m and 9m) and the primary outcome (12m) for DRI ($\rho _{3m,12m}=0.58$, $\rho _{6m,12m}=0.78$ and $\rho _{9m,12m}=0.89$; c.f. $\rho ^{*}_{s,t}=0.5$), meaning that at interim analysis inferences based on the modelling approach, that used all the data, gave much better estimates of the true end of trial treatment effect, than simple differences in primary outcome (12m) group means. The stronger than expected correlations also meant that the interim analyses generally occurred early than expected. That is, the observed number of participants providing 3m, 6m, 9m and 12m outcome data at each interim analysis were less than the expected number.

### FASHION

Two interim analyses were planned for the *simulated* group-sequential FASHION trial. The recruitment and follow-up profiles for FASHION were unusual (see Fig. 1), and reflected the phased approach to the study with an initial feasibility/pilot stage at a small number of sites, followed by more rapid recruitment as many more sites were opened. This resulted in relatively little benefit available from the early outcomes at the interim analyses; e.g. at the first interim analysis 12m data were available from $N_{12m}=62$ participants and 6m data from $N_{6m}=86$ participants. Of the four boundary settings tested, the FASHION trial would not have stopped at either interim analysis for futility or efficacy. Although at the second interim analysis there was emerging evidence, from the estimated treatment effect, indicating a likely benefit for the surgical treatment. However, this analysis occurred towards the end of recruitment, therefore the benefits of stopping so late would have been small. The reason for the late second interim analysis was the larger than expected value for the standard deviation of the 12m outcome; $\sigma _{12m}\approx 27$ rather than $\sigma ^{*}_{12m}=20$.

### CSAW

Two interim analyses were planned for the *simulated* group-sequential CSAW trial. For all four boundary settings tested, the CSAW study would not have stopped at the interim analyses, when data were available from $N_{6m}=79$ and $N_{6m}=137$ participants with 6m outcomes; no early outcomes were available for CSAW. At these interim analysis $N=142$ and $N=195$ participants had been recruited into the study and follow-up would have been completed in 21 and 28 months respectively; this compares to $N=210$ and 39 months for the original study. The expected standard deviation of the primary outcome ($\sigma ^{*}_{6m}=9$), used in the original sample size calculation, and used to build the group-sequential design, was smaller than the observed value ($\sigma _{6m}=12$) at the interim analyses. This caused the interim analyses to take place much later than planned (i.e. with more participants with 6m outcomes than expected; $N_{6m}=79$ and $N_{6m}=137$ rather than the expected $N^{*}_{6m}=40$ and $N^{*}_{6m}=80$). Given the small, but not clinically significant, result observed in the original study, it seems unlikely that any sensible stopping rule would have caused the CSAW study to stop early for efficacy, unless perhaps some early outcome data (e.g. 3m) had been available.

### FIXDT

Two interim analyses were planned for the *simulated* group-sequential FIXDT trial. For three of the four boundary settings tested, the FIXDT study would have stopped for futility, a result consistent with the original study, at the second interim analysis, when data where available from $N_{6m}=146$ participants with 6m outcomes, and $N_{3m}=178$ with 3m outcomes. At this interim analysis $N=243$ participants had been recruited into the study and follow-up would have been completed in 27 months; this compares to $N=321$ and 42 months for the original study. The estimated treatment effect ($\beta _{6m}$) for the 6m DRI outcome at the second interim analysis was -2.8 (95%CI; -10.1 to 4.4) favouring the control treatment (and -6.2 for the overrunning analysis); the estimate of the treatment effect in the original (fixed design) study was -4.0 (95%CI; -9.6 to 1.6). At the second interim analysis when the study was stopped, the estimated treatment effect was -2.8 (favouring the Nail), and the raw difference between groups was -0.5 (favouring the Nail). There was a strong correlation between the early outcome (3m) and the primary outcome (6m) for DRI ($\rho _{3m,6m}=0.65$). The expected standard deviation of the primary outcome ($\sigma ^{*}_{6m}=20$) used in the original sample size calculation, and used to build the group-sequential design, was a marked underestimate of the true value ($\sigma _{6m}=24$). This caused the study to be underpowered and the interim analyses to be at later times than planned (i.e. with more participants than expected).

### TOPKAT

Figure 1 shows the observed number of participants recruited and followed-up at 2 months, 1, 2, 3, 4 and 5 years for TOPKAT. The recruitment for TOPKAT completed in June 2013 and the first 5-year (primary) outcome data were not available until March 2015. Therefore, the window of opportunity, between some 5y final outcome data being available and recruitment completion was non-existent and as such the methodology we are investigating here, assessing possible early stopping of the trial based on using final outcome data before recruitment is completed, could not be used.

## Discussion

### Overview

For five of the selected surgical trials discussed here (WOLLF, FIXDT, DRAFFT, FASHION and WAT), the methodology of Parsons et al. [10], that used early outcome data in addition to final outcome data to inform stopping decisions at interim analyses, proved to be feasible. All of the putative group sequential designs described for these five studies used only information that was known (or thought to be known) or could have reasonably been speculated on (e.g. the numbers and patterns of patient data), at the study design and planning stage. The designs described here do not knowingly use any information from the observed trial publications or data. For this reason, we believe the results of the simulated group sequential trials, which used the observed data and the known dates when data were collected for each trial, are a true test of whether the design would have been possible, whether the trial would have stopped early and if so whether the result would have been consistent with that obtained from the original (fixed) design. The CSAW and TOPKAT studies were different from the other trials discussed here in two key respects that made an adaptive design of the type under discussion here impossible, the lack of early outcome data for CSAW and the lack of a window of opportunity for TOPKAT. For these reasons these two trials are discussed after the other five studies.

### WOLLF, FIXDT, DRAFFT, FASHION and WAT

Looking at each of these five trials in turn, the WOLLF study would have stopped early at the second interim analysis for three of the four boundary settings tested, when 293 participants had been recruited into the study (c.f. 460 in the original trial) and follow-up would have been completed in 27 months (c.f. 50 months for the original study). Inferences for the stopped studies would have been very similar to the original study; the analysis gave an estimate of the treatment effect equal to $-2.8$ (95%CI; $-11.4$ to 5.9) (favouring the control), whereas the treatment effect estimate in the original (fixed design) study was $-3.1$ (95%CI; $-8.5$ to 2.2). Also, of particular note in WOLLF were the extremely strong correlations between the early outcomes (3m, 6m and 9m) and the primary outcome (12m) for DRI. The strong correlations are important for two reasons. First, they allowed the modelling approach, that used all the data, to give better (more precise) estimates of the true end of trial treatment effect, than simple between group mean differences for the primary 12m outcome. And second, the stronger than expected correlations also meant that information accrued rapidly causing the interim analyses to occur earlier than might have been expected based on the number of participants with 12m outcomes alone. At the second interim analysis when the study was stopped, the estimated treatment effect from the model was $-2.8$ (favouring the control). In marked contrast, the raw difference between groups using 12m data only was 4.1 (conversely favouring NPWT). As we note above, the strong correlations meant that the model estimate was a much better estimate of the true treatment effect than simple between group difference in 12m outcomes. However, it is worth considering how this might have played out if this had been the situation in the real trial. Would the trial data monitoring and safety committee (DSMC) have had the confidence in the model estimate to stop the study for futility, when the difference in the means for 12m data alone were strongly favouring NPWT? The FIXDT trial was of a similar overall design to WOLLF, as it ran concurrently with and was designed by the same research team. As for WOLLF, for three of the four boundary settings tested, the FIXDT study would have stopped at the second interim analysis, when 243 (c.f. 321 in the original study) participants had been recruited into the study and follow-up would have been completed in 27 months (c.f. 42 months for the original study). Therefore, as for WOLLF, there would have been a considerable saving in time and cost, if an adaptive design had been used. The treatment estimate for FIXDT for the stopped study was $-2.8$ (95%CI; $-10.1$ to 4.4) favouring the control (Nail) treatment and the estimate of the treatment effect in the original (fixed design) study was $-4.0$ (95%CI; $-9.6$ to 1.6). The expected value of the standard deviation of the primary outcome ($\sigma ^{*}_{6m}=20$) used in the original sample size calculation, and used to build the group-sequential design, for FIXDT was a marked underestimate of the true value ($\sigma ^{*}_{6m}=24$). This caused the original study to be underpowered and the interim analyses to be at later times than planned. That is, when there were more participants than expected (146 versus 100) with 6m outcome data. WOLLF and FIXDT both reported results in the original studies favouring the control treatments; intramedullary nail fixation in FIXDT and standard dressing in WOLLF. The boundaries for our designs reflected the wish to stop for futility if there were a lack of emerging evidence to support better outcomes for the comparator test treatments (locking-plate fixation and NPWT respectively). Locking-plate fixation and NPWT both proved unlikely to be cost-effective compared to intramedullary nail fixation and a standard dressing respectively in reported health economic analyses [46, 47]. In contrast to WOLLF and FIXDT, the DRAFFT trial provided treatment effects throughout the study that tended to marginally favour the test (locking-plate) treatment over the control (wire fixation) treatment. Therefore, it is perhaps not surprising given the asymmetry of the boundaries that the DRAFFT trial would not have stopped at the interim analysis for any of the four boundary settings tested in the simulated studies. The estimate of the standard deviation of the primary outcome for DRAFFT ($\sigma _{12m}$), used in the original sample size calculation and to build the group-sequential design, was much smaller than expected (13.5 versus 20). This caused the interim analysis to take place much earlier than planned when there were many fewer participants with 12m outcomes than expected; 26 rather than the expected 100. The stronger than expected correlations also in part contributed to the early interim analysis, as did the extremely rapid recruitment to DRAFFT caused by a surge in recruitment due to the harsh Winter weather causing a surge in distal radius fracture as a consequence of falls. However, given the consistent positive treatment effects in favour of the plate intervention it seems unlikely that better estimates of the covariance parameters or change of boundaries (within reason) would have caused the study to stop early for futility. The FASHION study had a quite different recruitment profile from the other trials, due to the phased approach with an initial feasibility/pilot stage at a small number of sites, followed by more rapid recruitment as sites were opened. This caused there to be relatively little benefit available from early outcomes (6m) in addition to that provided by the final 12m outcomes. Of the four boundary settings tested, the FASHION study would not have stopped at either interim analysis for futility or efficacy. The two interim analyses both provided evidence in favour of the surgical intervention, but test statistics were not of sufficient magnitude to cause the trial to stop. Although, the estimated treatment effect after completing follow-up for the second interim analysis (overrunning analysis) was consistent with result of the original study, which reported a positive result in favour of the surgery intervention. The lack of stopping (for efficacy) for FASHION is in large part due to the asymmetric selection of boundaries that made it relatively hard to stop for efficacy. The boundaries used here reflect the view, that we suspect is widespread amongst T&O trialists, that much stronger evidence is required to cause a trial to stop for efficacy than futility. Many T&O clinicians believe that if there is emerging evidence for efficacy then a trial should complete recruitment to target in order to provide a precise estimate of the treatment effect and capture as much safety information as possible (e.g. adverse events). Given the relatively small sample size and the small (clinically unimportant) result observed in the original WAT trial, it seems unlikely that any sensible stopping rule would have caused the study to stop early. For all four boundary settings tested, the WAT study would not have stopped at the interim analysis.

### CSAW and TOPKAT

For the CSAW and TOPKAT trials it was not possible to use the methodology of Parsons et al. [10] directly as for the former study there were no early outcome data available and for the latter no final outcome data were available prior to recruitment completing. Therefore, for TOPKAT we did not proceed to simulate an adaptive study based on early assessment of the treatment effect on the final outcome. For CSAW, although no early outcome data were available, we did simulate how the study would have proceeded via a group sequential design based simply on the final outcome data alone. For the other trials examined here, this would have been equivalent to using the test statistic *Z*0, rather than *Z*, to make stopping decisions. Using this methodological approach, there is no evidence in the simulated study to believe that an adaptive design would have caused CSAW to stop early.

## Conclusion

The results for five of the studies reported here (WOLLF, FIXDT, DRAFFT, FASHION and WAT) showed that adaptive design using early outcome data would have been feasible and likely to provide designs that were at least as efficient, and possibly more efficient, than the original fixed sample size designs. For WOLLF and FIXDT the simulations showed that it was highly likely these studies would have (correctly) stopped early for futility, saving potential considerable effort and resources. WOLLF particularly showed the important part that early outcome data particularly can play, as analyses based purely on the final outcome data alone would have meant that stopping (for any reason) would have been unlikely. The boundaries selected here favoured stopping for futility, at the cost of making stopping for efficacy unlikely, unless there were very strong evidence available. For this reason, the two studies that showed modest effect estimates at interim analyses in favour of the test treatment (WAT and DRAFFT), did not stop early. This was consistent with the final results of these studies. The FASHION trial showed good evidence in favour of the test surgical intervention in the final analysis but fell short of stopping at the interim analyses. For this study it would have been possible to select different, but sensible, boundaries that would have resulted in early stopping for efficacy. For all the studies it was clear that the feasibility and practicality of using the methods proposed by Parsons et al. [10] was determined in large part by (i) the width of the window of opportunity for stopping, (ii) the available of early outcome data and their correlations with final outcomes, (iii) recruitment and outcome data follow-up (FU) accrual profiles and (iv) the veracity of the estimates of the covariance parameters available at the design planning stage. The first of the three issues we highlight here were evident for all the trials. If there were little or no final outcome data available at interim analyses, and little or no early outcome data were available or uncorrelated with final outcomes then decision making for early stopping was simply not possible. The pattern of data accrual and follow-up were important determinants of the feasibility of the methods used. However, more work is needed to fully understand the impact of different approaches to recruitment and FU on the widespread applicability of the methods. For instance, it seems possible that limiting or increasing recruitment at certain stages of a trial (e.g. by delaying or bring forward initiation of new recruitment centres) may be beneficial in certain circumstances. It was also clear for a number of the trials that the times when interim analyses occurred were either much earlier or later than expected. This was largely due to estimates of the covariance estimates used in the initial planning being markedly different from the observed values. For instance, if correlations between outcomes were stronger than expected and variances smaller, then interim analyses would occur sooner than might have been expected. This in itself is not necessarily problematic, as we deliberately motivated stopping based on information rather than purely sample size considerations. However, in instances where interim analyses occurred particularly early (e.g. in the DRAFFT study an interim analysis occurred when there were final outcome data from 26 participants rather than the expected 100), it is likely that in practice it would have been difficult for the DSMC, trial management group and trial steering committees (TMG & TSC) to make and confirm stopping decisions and justify these to the funding body based on so few data. In practice, either minimum sample sizes might have to be pre-specified or interim analyses plans be modified as the study proceeds (e,g, by using blinded re-estimation of the covariance parameters as data accumulates to update the trial plans). In many of the trials, correlations between outcomes were much stronger than expected (e.g. $> 0.7$). If there are such strong correlations between early and final outcomes, then it may also be that there are strong correlations between treatment effect estimates (e.g. treatment effects for an early outcome at 6 months were much the same as those for the primary outcome at 12 months). If this is the case, then arguably we might want to consider using the 6 months outcome as the primary. If this is the case, then this would be a simpler strategy to shorten the trial and save costs. Clearly, for a number of the trials expected values of standard deviations ($\sigma ^{*}_{t}$) were considerably different from observed values, and this caused interim analyses to occur somewhat earlier or later than planned. In practice, to implement the methodology described here, we would ideally like good estimates of $\sigma _{t}$ at the planning stage. However, we do not see this a problem particular to group sequential designs as poor estimates of $\sigma _{t}$ would have equally detrimental effects on conventional (fixed) designs (e.g. sample sizes from power calculations). As the method we describe is based on the use of the alpha spending function approach to control the type I error rate [11, 20], there is flexibility over the timing of the interim analyses, although in our study we have not exploited this option. Deviation from the planned interim analysis timing will have an impact on trial power. This is true for the alpha spending function approach in more conventional settings (with a single primary outcome), but is yet to be evaluated in this setting. We plan to explore this and a number of other issues around the timing of interim analyses and follow-up in future simulation work. The results of the overrunning analyses, that used complete follow-up data for all participants recruited into a trial, did not generally differ much, in terms of inferences (i.e. qualitatively), from the analyses that used only the data available at the interim analyses. The additional time to complete follow-up could be as much as a year in some studies (e.g. WOLLF, DRAFFT, FASHION, WAT). Although the overrunning analyses resulted in improved precision in the treatment effect estimates this should be balanced against the need to report results in a timely and ethically sound manner (to stop patients receiving possibly harmful or ineffective treatments). Therefore, although we acknowledge the need to complete follow-up on all participants recruited into a study, the decision about whether to report analyses immediately using the data available when the trial stopped or wait to complete follow-up will be trial dependent and should be agreed on prior to commencement by all stakeholders. Also, it is of course always important to not only consider the potential benefits but also the pitfalls of stopping a trial early. For instance, stopping a study early for futility will in principle reduce resource use and costs. However, this benefit may be negligible (i.e. unimportant) in trial settings where recruitment sites remain open and patients remain on study treatments after the trial stops [48]. The focus of the work described here has been primarily on futility stopping (e.g. choosing the four alternate futility boundaries). This was due to nature of the selected examples which (reflecting our own interests) were all pragmatic publicly funded trials testing licensed, but otherwise untested, interventions where early stopping caused by a lack of evidence for efficacy was of primary interest. However, if different choices of example trials had been made then we could just have easily have made analogous arguments for stopping early for efficacy, using the same general methodological approach.

There are a number of limitations to this study. We have tried, as much as possible, to avoid using information that was only known after trial data were available when planning the adaptive designs. For instance, by using estimates of variances from the published protocols and, where possible, details of recruitment and follow-up strategies from the trial teams. However, it may be that the results or knowledge of the selected trials may have unconsciously influenced the adaptive designs (e.g. the timing and number of interim analyses). We have used the date when outcome data were ‘collected’ as a proxy for when it would have been available to make stopping decisions. However, in reality in a trial it often takes some time to enter the data on the study database and extract data (e.g. freeze and check data ready for analysis). These data would then need to be sent to the trial statistician to undertake an analysis, circulate to colleagues on a DSMC to meet and discuss the results and make a recommendation to the TMG/TSC to finalize the decision. This would typically take some time - a number of weeks at least. We have not accounted for these delays in the simulation study, so it maybe that our assessments of the savings an adaptive design might have made (in terms of time and cost) may be somewhat optimistic. Although, in reality many of these tasks could be better planned, streamlined and automated to some extent, if an adaptive design were being used.

## Supplementary Information

**Additional file 1:** Seven recent surgical randomized controlled trials: descriptions and results of retrospective analysis using group sequential designs.

**Additional file 2:** Group sequential designs for longitudinal outcomes.

## Data Availability

The data summaries and code written as part of this study are available from the corresponding author on reasonable request.

## Abbreviations

EME: (UK) Efficacy and Mechanism Evaluation programme
MRC: (UK) Medical Research Council
NIHR: (UK) National Institute of Health Research
START:REACTS: Sub-acromial spacer for Tears Affecting Rotator cuff Tendons: a Randomised, Efficient, Adaptive Clinical Trial in Surgery
WAT: Warwick Arthroplasty Trial
DRAFFT: Distal Radius Acute Fracture Fixation Trial
WOLLF: Wound management of Open Lower Limb Fractures study
FASHION: Full UK RCT of Arthroscopic surgery for Hip Impingement versus best cONservative care study
CSAW: Can Shoulder Arthroscopy Work trial
FIXDT: FIXation of Distal Tibia fractures trial
TOPKAT: Total Or Partial Knee Arthroplasty Trial
DRI: Disability Rating Index
PRWE: Patient Rated Wrist Evaluation
OHS: Oxford Hip Score
OSS: Oxford Shoulder Score
OKS: Oxford Knee Score
iHOT-33: International Hip Outcome Tool
DSMC: Data Safety and Monitoring Committee
TSC: Trial Steering Committee
TMG: Trial Management Group
CI: Confidence Interval
FU: Follow-up
PROM: Patient-reported Outcome
MCID: Minimum Clinically Important Difference
RCT: Randomized Controlled Trial
T&O: Trauma and Orthopaedics
