# Supplementary material for: Group sequential designs in pragmatic trials: feasibility and assessment of utility using data from a number of recent surgical RCTs
Source: BMC Med Res Methodol. 2022 Oct 1;22:256. doi: 10.1186/s12874-022-01734-2 (PMC9526271; doi:10.1186/s12874-022-01734-2)
Supplement: Supplementary file 1 — Additional file 1: Seven recent surgical randomized controlled trials: descriptions and results of retrospective analysis using group sequential designs. [file 12874_2022_1734_MOESM1_ESM.pdf]

Seven recent surgical randomized controlled trials: descriptions and results of retrospective analysis using group sequential designs

Nick R Parsons[1], Nigel Stallard[1], Helen Parsons[2], Aminul Haque[2], Martin Underwood[2,3], James Mason[2], Iftekhar Khan[2], Matthew L Costa[4], Damian R Griffin[2], James Griffin[2], David J Beard[4], Jonathan A Cook[5], Loretta Davies[4], Jemma Hudson[6], and Andrew Metcalfe[2,3]

[1]Statistics and Epidemiology Unit, Warwick Medical School, University of Warwick, CV4 7AL, Coventry, UK
[2]Warwick Clinical Trials Unit (WCTU), Warwick Medical School, University of Warwick, CV4 7AL, Coventry, UK
[3]University Hospital Coventry and Warwickshire (UHCW), CV2 2DX, Coventry, UK
[4]Nuffield Department of Orthopaedics, Rheumatology and Musculoskeletal Sciences (NDORMS), University of Oxford, OX3 7LD, Oxford, UK
[5]Centre for Statistics in Medicine, Nuffield Department of Orthopaedics, Rheumatology and Musculoskeletal Sciences (NDORMS), University of Oxford, OX3 7LD, Oxford, UK
[6]Health Services Research Unit (HSRU), University of Aberdeen, AB25 2ZD, Aberdeen, UK

September 27, 2022

1 Introduction

The following sections (2 to 8) consist of a brief description of seven recent surgical randomized controlled trials (RCTs), all in the clinical specialty of trauma and orthopaedics. The RCTs are all conventional two-arm parallel group fixed sample size designs, chosen because they all followed-up participants at a number of fixed time-points (t), using the same outcome measure at each time-point, with one time-point being the pre-specified primary (final) end-point and others classified as either early or late end-points. For each RCT a plan for a group sequential adaptive design is formulated, based on the information available in the published protocols and what could have plausibly been known by the trial team prior to the trial commencing recruitment. A number of interim analyses are planned for each RCT based on reaching an information threshold (I), with binding stopping rules characterized by upper and lower boundaries for a test statistic Z determined by the data available at the interim analyses. The test statistic at follow-up time-point t is given by $Z = \beta_t/\mathrm{sd}(\beta_t)$, where β_t is the treatment effect at time t estimated by fitting a longitudinal model using all the data available from both early and final end-points at the interim analysis. As a comparator, effect estimates and test statistics based on final outcome data only are also available, and are labelled as $\beta0_t$. A full description of the methods used to monitor information accrual and estimate treatment effects β_t and $\beta0_t$ and variances and example code in R for implementation are provided in *Group sequential designs for longitudinal outcomes* [Additional file 2]. For each RCT information accrual during recruitment is monitored using the observed trial data and dates when data were available on the trial databases, and interim analyses undertaken in order to simulate whether the trials might have stopped early, if an adaptive design had actually been used. For each RCT data, treatment effect and covariance parameter estimates and stopping decisions are provided together with a summary of the stopping decisions.

2 The Warwick Arthroplasty Trial (WAT)

2.1 Details

The Warwick Arthroplasty Trial (WAT) was a single centre, two arm, parallel group, assessor blinded, randomised controlled trial with a 1:1 treatment allocation conducted in the UK [1, 2], recruiting $N = 126$ patients aged 18

years and over who were medically fit for an operation, and suitable for a resurfacing arthroplasty of the hip; Figure 1 shows the progress of recruitment and followed-up (FU) at 6 weeks, 3, 6 and 12 months for WAT. Patients were recruited between May 2007 and February 2010 from hip replacement clinics at the University Hospitals Coventry and Warwickshire NHS trust (Coventry, UK.) and randomly assigned to receive either a total hip arthroplasty (THA) or a resurfacing arthroplasty (RSA). The primary outcome was hip function, as measured by the patient-reported Oxford Hip Score (OHS) at 12 months (12m) after operation, with early outcome assessed at 6 weeks (6w), 3 months (3m) and 6 months (6m). The OHS provides a score on a scale from 0 to 48, with 48 representing no pain and perfect function, and lower scores indicating pain and poor function.
The primary result of the trial was that there was no statistically significant difference in OHS at 12 months; the mean score in the RSA group was 40.4 (with $N1_{12m} = 57$ participants providing 12 month data) and in the THA group 38.2 ($N0_{12m} = 63$), a difference of 2.23 (95%CI; -0.51 to 12.58) (see Table 2 in main clinical paper [2]).

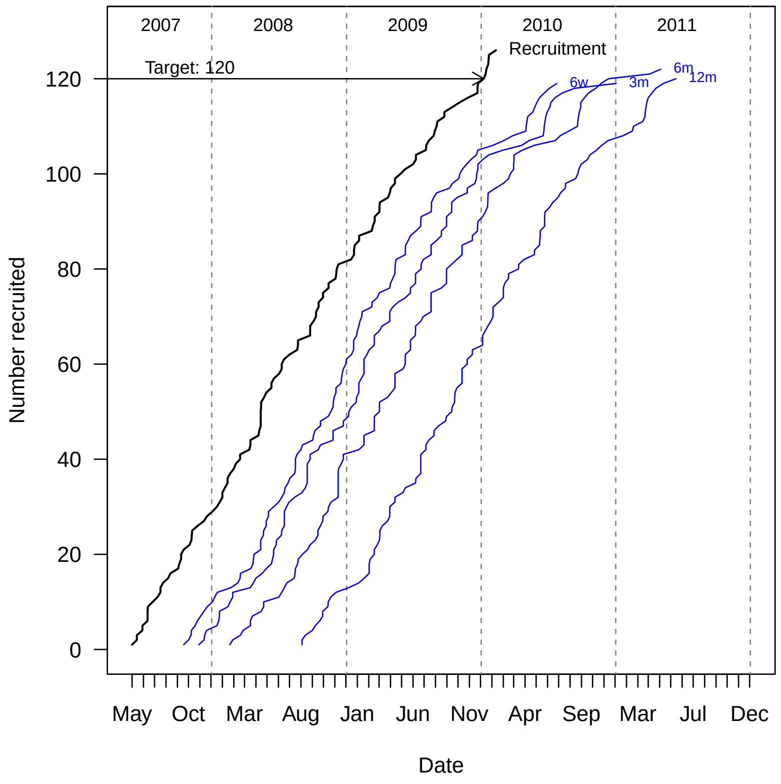

Figure 1: Progress of recruitment (—) and follow-up (—) for WAT

2.2 Group sequential adaptive design

The trial reported early outcomes for OHS at 6w, 3m and 6m, with the primary outcome at 12m. From the WAT trial protocol, the planned sample size was $N = 104$ for 80% power, based on standard deviation at 12 months $\sigma_{12m} = 9$ and minimum clinically important difference (MCID) of 5 for OHS [1]. Allowing 10% loss to FU, gave $N = 120$. For the selected trial interventions, there is often a long wait from recruitment until the operation takes place. Therefore, outcomes during follow-up were also expected to often be later than would have been anticipated from the recruitment date alone. For instance, if the operation took place 2 months after recruitment, the 6w, 3m, 6m and 12m follow-ups, which were based on the operation date, were consequently delayed by 2 months. This results in a relatively narrow window of opportunity for interim analyses (the time interval between final outcome data becoming available and the end of recruitment) and therefore it seems likely that one interim analysis would have been the most likely option to select at planning.

If recruitment had proceeded to target as originally planned, and we assume that $N0^*_{12m} = N1^*_{12m} = 52$, then the information at the trial end would be given by $I^*_{End} = 26/9^2 = 0.321$; we use the $*$ superscript purely as a notational convenience to indicate expected values (based on our assumptions) rather than the values observed in the trial itself. Inspection of the planned recruitment schedules for the trial suggests that one interim analysis would have been feasible for WAT. Assume now, for the purposes of planning the interim analysis, that numbers in the intervention arms are always equal (i.e. $N0^*_t = N1^*_t$) and the overall sample size is $N^*_t = N0^*_t + N1^*_t$), and that the interim analysis could have feasibly occurred when $N^*_{12m} = 40$. The expected numbers of early outcomes at the interim analysis could have been as follows, based on the patterns of follow-up that might have been expected before recruitment started:

Interim	N^*_{6w}	N^*_{3m}	N^*_{6m}	N^*_{12m}
1	80	70	60	40

Assuming that the standard deviations are the same for the early outcomes as for the final outcome $\sigma^*_{6w} = \sigma^*_{3m} = \sigma^*_{6m} = \sigma^*_{12m} = 9$, and that the correlations between outcomes are such that $\rho^*_{6w,3m} = \rho^*_{6w,6m} = \rho^*_{6w,12m} = \rho^*_{3m,6m} = \rho^*_{3m,12m} = \rho^*_{6m,12m} = 0.5$, then the information required at the interim analysis is as follows, $I^*_1 = 0.150$ (see *Group sequential designs for longitudinal outcomes* [Additional file 2] for details of calculation and implementation). Using the following four options for futility (cumulative) stopping probabilities, (a) $\alpha^*_l = (0.160, 0.975)$, (b) $\alpha^*_l = (0.320, 0.975)$, (c) $\alpha^*_l = (0.480, 0.975)$ and (d) $\alpha^*_l = (0.640, 0.975)$ and efficacy stopping probabilities (for all options) $\alpha^*_u = (0.005, 0.025)$, gives the following lower (l_a, l_b, l_c, l_d) and upper (u_a, u_b, u_c, u_d) stopping boundaries for the test statistic (Z) at each interim analysis and the trial end:

Design		1	End		1	End
(a)	l_a	-0.99	2.01	u_a	2.58	2.01
(b)	l_b	-0.47	2.01	u_b	2.58	2.01
(c)	l_c	-0.05	2.00	u_c	2.58	2.00
(d)	l_d	0.36	1.98	u_d	2.58	1.98

These stopping boundaries were used to simulate the progress of the trial and assess whether the trial would have stopped at the interim analysis.

2.3 Results

The numbers of observed trial participants providing data at the interim analysis and the trial end is shown in Table 1 and the total numbers of participants recruited at the time the interim analysis took place is shown in Table 4.

Table 1: Numbers of observed and expected participants providing 6w, 3m, 6m and 12m outcome data at the interim analysis and the trial end for WAT.

Interim	Observed				Expected			
	N_{6w}	N_{3m}	N_{6m}	N_{12m}	N^*_{6w}	N^*_{3m}	N^*_{6m}	N^*_{12m}
1	49	43	29	10	80	70	60	40
End	119	119	122	120	104	104	104	104

Estimated treatment effects at 12m, from the longitudinal model exploiting early outcome data (β_{12m}) and using final outcome data only ($\beta0_{12m}$), test statistics (Z and $Z0$) and information thresholds used to trigger interim analysis I^* and observed information $I = 1/\text{var}(\beta_{12m})$ (which will always be $\geq I^*$ at interim analysis) are shown in Table 2.

Table 3 shows the estimated correlations and standard deviations at the interim analysis and Figure 2 shows scatter plots of the observed data between time-points. The estimated standard deviations for the 12-month outcome at the interim analysis was much smaller than expected; σ_{12m} was expected to around 9 but was actually nearer to 5. Also, the correlations were generally larger than expected. These factors explain why the numbers of participants at the interim analysis was much smaller than planned, as it took many fewer participants to accumulate the required information, due to the larger than expected correlations and smaller than expected value for σ_{12m}.

Table 2: Means and estimates of treatment effects at the interim analysis and the trial end for WAT.

Interim	$m1_{12m}$	$m0_{12m}$	Δ	β_{12m}	var(β_{12m})	Z	I	I^*	$\beta0_{12m}$	var($\beta0_{12m}$)	$Z0$
1	45.00	38.20	6.80	4.30	6.58	1.68	0.152	0.150	6.80	14.64	1.78
End	40.40	38.17	2.23	2.18	3.54	1.16	0.283	0.321	2.23	3.59	1.18

$m0_{12m}$ and $m1_{12m}$ are the means for the THA (control) and RSA arms and $\Delta = m1_{12m} - m0_{12m}$, and $\Delta = \beta0_{12m}$

Table 3: Estimates of correlations and standard deviations at the interim analysis and the trial end; the design model assumed $\rho^* = 0.5$, for all pairs of times and $\sigma^* = 9$ for all times for WAT.

Interim	$\rho_{6w,3m}$	$\rho_{6w,6m}$	$\rho_{6w,12m}$	$\rho_{3m,6m}$	$\rho_{3m,12m}$	$\rho_{6m,12m}$	σ_{6w}	σ_{3m}	σ_{6m}	σ_{12m}
1	0.86	0.80	0.60	0.90	0.71	0.50	9.93	10.43	9.36	5.58
End	0.80	0.69	0.59	0.83	0.72	0.80	10.06	9.84	8.95	10.37

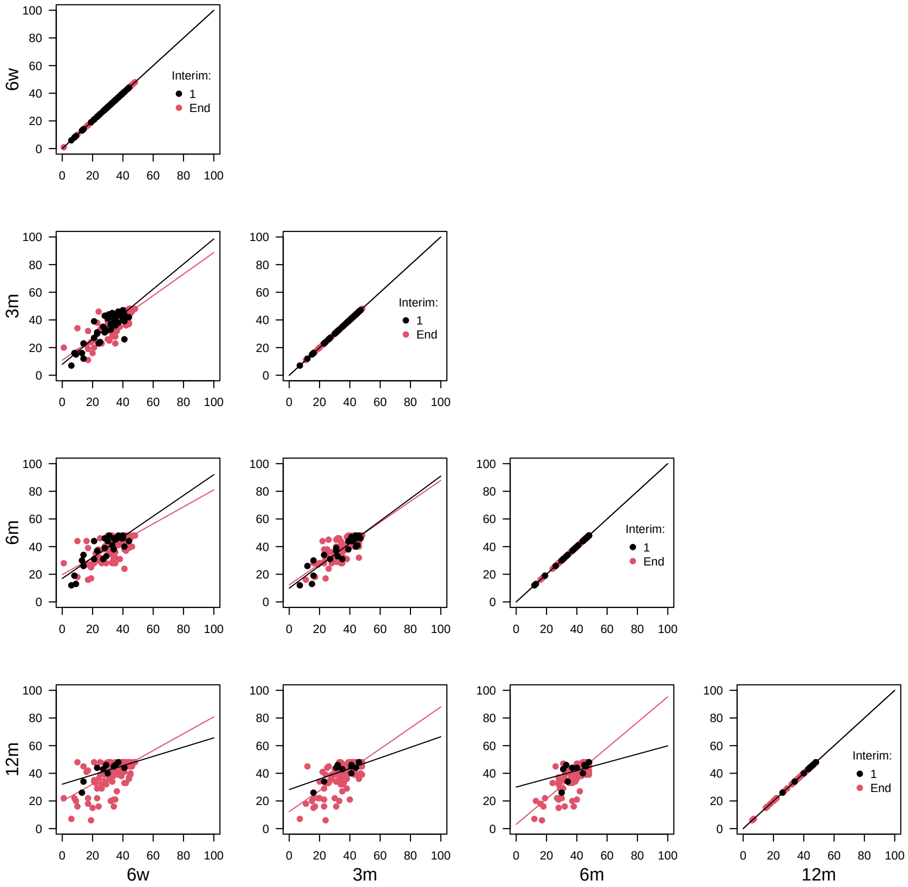

Figure 2: Scatter plots of the observed data between time-points, with fitted lines, for the interim analysis and trial end for WAT.

Test statistic Z is in the continuation region (between upper and lower boundaries) at the interim analysis, for all boundary settings (a) to (d).
Looking at the overrunning analysis (that includes the complete follow-up data on participants recruited into the trial at the interim analysis), gives the numbers of trial participants providing data at each interim analysis shown in Table 4 and estimated model parameters and test statistics in Table 5.

The test statistics (Z) from Table 5 confirm the results of the stopping decisions based on the interim data, and indicate that the trial would not have stopped for any of the settings (a) to (d).

Table 4: Numbers of participants providing 3w, 3m, 6m and 12m outcome data at each overrunning analysis, and the total trial recruitment (N) and trial length (in months) for WAT.

Interim	N_{6w}	N_{3m}	N_{6m}	N_{12m}	N	Months
1	73	72	73	73	75	17
End	119	119	122	120	126	48

Table 5: Means and estimates of treatment effects at each overrunning analysis and the trial end for WAT.

Interim	$m1_{12m}$	$m0_{12m}$	Δ	β_{12m}	var(β_{12m})	Z	I	$\beta0_{12m}$	var($\beta0_{12m}$)	$Z0$
1	40.80	37.45	3.35	3.34	6.0	1.35	0.164	3.35	6.10	1.36
End	40.40	38.17	2.23	2.18	3.54	1.16	0.283	2.23	3.59	1.18

$m0_{12m}$ and $m1_{12m}$ are the means for the THA (control) and RSA arms and $\Delta = m1_{12m} - m0_{12m}$, and $\Delta = \beta0_{12m}$

The test statistics (Z) from the overrunning analysis shown in Table 5 confirm the results of the stopping decisions based on the interim data (Table 2). In summary, the test statistics from the interim analysis in Table 2, and the stopping boundaries from Section 2.2, indicate that the WAT RCT would not have stopped at the interim analysis for any of the design settings.

3 The Distal Radius Acute Fracture Fixation Trial (DRAFFT)

3.1 Details

The Distal Radius Acute Fracture Fixation Trial (DRAFFT), trial compared Kirschner wire fixation (Wire) with volar locking plate fixation (Plate) for patients with a dorsally displaced fracture of the distal radius [3, 4]. The trial used the Patient Rated Wrist Evaluation (PRWE) score at 12 months (12m) after surgery to assess participant outcomes; PRWE measures a patient's experience of pain and disability to give a score out of 100 (with 100 being the worst score). The trial recruited $N = 461$ participants from July 2010 to July 2012, randomising $N0 = 230$ to the Wire group (control) and $N1 = 231$ to the Plate group, on a 1:1 basis. Early PRWE outcomes were assessed at 3 and 6 months; Figure 3 shows the progress of recruitment and followed-up at 3, 6 and 12 months for DRAFFT.
The primary result of the trial was that there was no statistically significant difference in PRWE score at 12 months; the mean score in the Wire group was 15.3 ($N0_{12m} = 211$) and in the Plate group 13.9 ($N1_{12m} = 204$), a difference of 1.4 (95%CI; -1.8 to 4.5) (see Table 3 in main clinical paper [4]). The trial took 34 months (recruitment plus follow-up) to complete.

3.2 Group sequential adaptive design

The DRAFFT trial recorded early PRWE outcome at 3m and 6m, with the primary outcome at 12m. From the DRAFFT trial [3], planned sample size $N = 350$, based on standard deviation $\sigma_{12m} = 20$ and MCID of 6 for PRWE. Allowing 10% loss to FU, this gave $N = 390$. As a result of faster than expected recruitment to the trial, and with the permission of the review board, the sample size was increased resulting in $N = 461$ participants. Recruitment to the trial was expected to be rapid, as this is a common injury (particularly in the winter months), therefore the window of opportunity for interim analyses would have been narrow, as recruitment could feasibly have been completed before any 12 months outcome data were available. For this reason, it seems likely that one interim analysis would have been the most likely option to select at planning.
If recruitment had proceeded to target as originally planned, and we assume that $N0^*_{12m} = N1^*_{12m} = 175$, then the information at the trial end would be given by $I^*_{End} = 87.5/20^2 = 0.219$. Inspection of the planned recruitment schedules for the trial suggests that only one interim analysis would have been feasible for DRAFFT. Assume now, for the purposes of planning the interim analysis, that numbers in the intervention arms are always equal, and that the interim analysis could have feasibly occurred when $N^*_{12m} = 100$. The expected numbers of early outcomes at the interim analysis could have been as follows, based on the patterns of follow-up that might have been expected before recruitment started:

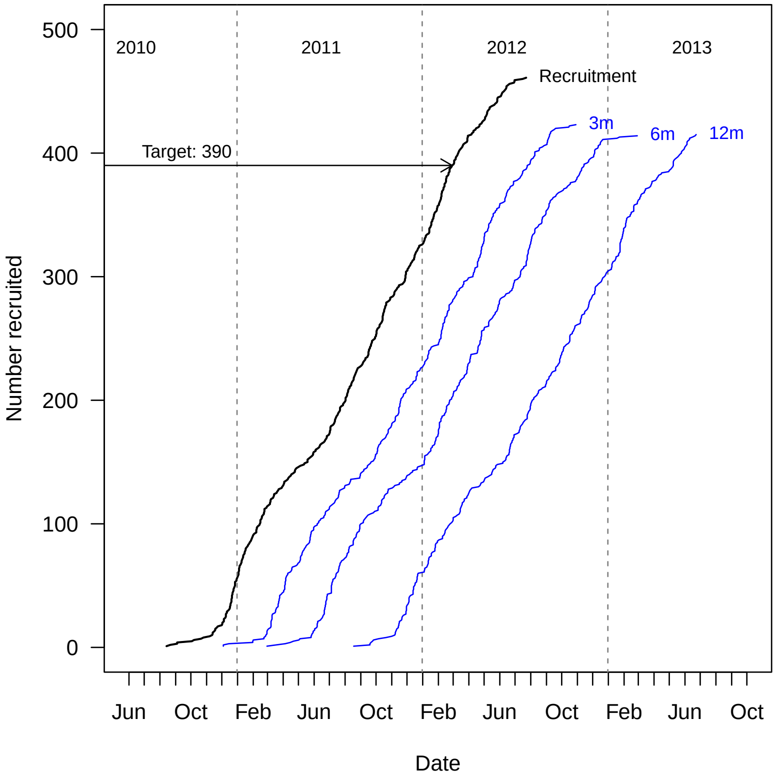

Figure 3: Progress of recruitment (—) and follow-up (—) for DRAFFT

Interim	N^*_{3m}	N^*_{6m}	N^*_{12m}
1	200	140	100

Assuming that the standard deviations are the same for the early outcomes as for the final outcome $\sigma^*_{3m} = \sigma^*_{6m} = \sigma^*_{12m} = 20$, and that the correlations between outcomes are such that $\rho^*_{3m,6m} = \rho^*_{3m,12m} = \rho^*_{6m,12m} = 0.5$, then the information required at the interim analysis is as follows, $I^*_1 = 0.073$. Using the four selected design options for futility (cumulative) stopping probabilities, (a) $\alpha^*_l = (0.160, 0.975)$, (b) $\alpha^*_l = (0.320, 0.975)$, (c) $\alpha^*_l = (0.480, 0.975)$ and (d) $\alpha^*_l = (0.640, 0.975)$ and efficacy stopping probabilities (for all options) $\alpha^*_u = (0.005, 0.025)$, gives the following lower and upper stopping boundaries for the test statistic (Z) at each interim analysis and the trial end:

Design		1	End		1	End
(a)	l_a	-0.99	2.02	u_a	2.58	2.02
(b)	l_b	-0.47	2.01	u_b	2.58	2.01
(c)	l_c	-0.05	2.00	u_c	2.58	2.00
(d)	l_d	0.36	1.97	u_d	2.58	1.97

These stopping boundaries were used to simulate the progress of the trial and assess whether the trial would have stopped at the interim analysis.

3.3 Results

The numbers of observed trial participants providing data at the interim analysis is shown in Table 6 and the total numbers of participants recruited at the time the interim analysis took place is shown in Table 9.

Table 6: Numbers of observed and expected participants providing 3m, 6m and 12m outcome data at the interim analysis and the trial end for DRAFFT.

Interim	Observed			Expected		
	N_{3m}	N_{6m}	N_{12m}	N^*_{3m}	N^*_{6m}	N^*_{12m}
1	205	135	26	200	140	100
End	423	414	415	350	350	350

Estimated treatment effects at 12m, from the longitudinal model exploiting early outcome data (β_{12m}) and using final outcome data only ($\beta0_{12m}$), test statistics (Z and $Z0$) and information thresholds used to trigger interim analysis I^* and observed information $I = 1/\text{var}(\beta_{12m})$ are shown in Table 7.

Table 7: Means and estimates of treatment effects at the interim analysis and the trial end for DRAFFT.

Interim	$m1_{12m}$	$m0_{12m}$	Δ	β_{12m}	$\text{var}(\beta_{12m})$	Z	I	I^*	$\beta0_{12m}$	$\text{var}(\beta0_{12m})$	$Z0$
1	12.29	16.68	4.39	1.41	12.54	0.40	0.080	0.073	4.39	24.42	0.89
End	13.93	15.30	1.37	1.51	2.59	0.94	0.387	0.219	1.37	2.60	0.85

$m0_{12m}$ and $m1_{12m}$ are the means for the Wire and Plate arms and $\Delta = m0_{12m} - m1_{12m}$, and $\Delta = \beta0_{12m}$

Table 8 shows the estimated correlations and standard deviations at the interim analysis and Figure 4 shows scatter plots of the observed data between time-points. The estimated standard deviations for the 12-month outcome are much smaller than expected when planning the trial; σ_{12m} was expected to around 20 but was actually nearer to 15. This explains why the numbers of participants at the interim analysis was much smaller than planned, as it took fewer participants to accumulate the required information, due to the smaller than expected value for σ_{12m}. Also, the fast rate of recruitment and small number of 12m outcomes meant that it was problematic to hit the required information level exactly; the value for I is considerably larger than I^* at the interim analysis.

Table 8: Estimates of correlations and standard deviations at the interim analysis and the trial end; the design model assumed $\rho^* = 0.5$, for all pairs of times and $\sigma^* = 20$ for all times for DRAFFT.

Interim	$\rho_{3m,6m}$	$\rho_{3m,12m}$	$\rho_{6m,12m}$	σ_{3m}	σ_{6m}	σ_{12m}
1	0.81	0.78	0.72	22.34	17.45	13.51
End	0.75	0.61	0.73	22.57	18.21	16.63

Test statistic Z is in the continuation region (between upper and lower boundaries) at interim analysis 1 for all boundary settings (a) to (d), indicating that the trial would not have stopped.
Looking at the overrunning analysis, gives the numbers of trial participants providing data at each interim analysis shown in Table 9 and estimated model parameters and test statistics in Table 10.

Table 9: Numbers of participants providing 3m, 6m and 12m outcome data at each overrunning analysis, and the total trial recruitment (N) and trial length (in months) for DRAFFT.

Interim	N_{3m}	N_{6m}	N_{12m}	N	Months
1	267	264	271	294	15
End	423	414	415	461	34

The test statistics (Z) from the overrunning analysis shown in Table 10 confirm the results of the stopping decisions based on the interim data (Table 7). In summary, the test statistics from the interim analysis in Table 7, and the stopping boundaries from Section 3.2, indicate that the DRAFFT RCT would not have stopped at the interim analysis for any of the design settings.

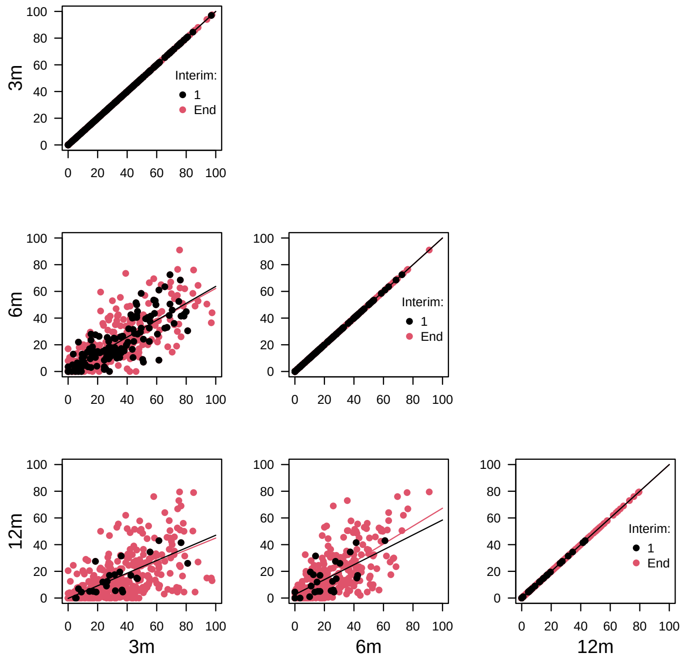

Figure 4: Scatter plots of the observed data between time-points, with fitted lines, for the interim analysis and trial end for DRAFFT.

Table 10: Means and estimates of treatment effects at each overrunning analysis and the trial end for DRAFFT.

Interim	$m1_{12m}$	$m0_{12m}$	Δ	β_{12m}	var(β_{12m})	Z	I	$\beta0_{12m}$	var($\beta0_{12m}$)	$Z0$
1	15.54	16.38	0.83	1.14	4.38	0.54	0.228	0.83	4.38	0.40
End	13.93	15.30	1.37	1.51	2.59	0.94	0.387	1.37	2.60	0.85

$m0_{12m}$ and $m1_{12m}$ are the means for the Wire and Plate arms and $\Delta = m0_{12m} - m1_{12m}$, and $\Delta = \beta0_{12m}$

4 The Wound management of Open Lower Limb Fractures (WOLLF) trial

4.1 Details

The Wound management of Open Lower Limb Fractures (WOLLF) trial was a multi-centre randomized trial performed in the UK Major Trauma Network, recruiting $N = 460$ patients aged 16 years or older with a severe open fracture of the lower limb from July 2012 to December 2015 [5, 6]. The main objective of the trial was to assess the disability of patients with a severe open fracture of the lower limb treated with either negative pressure wound therapy (NPWT) or standard (Standard) wound management after the first surgical debridement of the wound. The primary outcome of the trial was the Disability Rating Index (DRI) score (range, 0 = no disability to 100 = completely disabled) at 12 months (12m), with early outcomes measured at 3, 6 and 9 months. The trial recruited $N = 460$ participants who were randomised in a 1:1 ratio to either NPWT ($N1 = 226$) or Standard ($N0 = 234$) wound treatment; Figure 5 shows the progress of recruitment and followed-up (FU) at 3, 6, 9 and 12 months for WOLLF.

The primary result of the trial was that there was no statistically significant difference in DRI score at 12 months; the mean score in the NPWT group was 45.5 ($N1_{12m} = 179$) and in the standard dressing group 42.4 ($N0_{12m} = 195$), a difference of -3.1 (95%CI; -8.5 to 2.2) (see Table 2 in main clinical paper [6]). The trial took 50 months (recruitment plus follow-up) to complete.

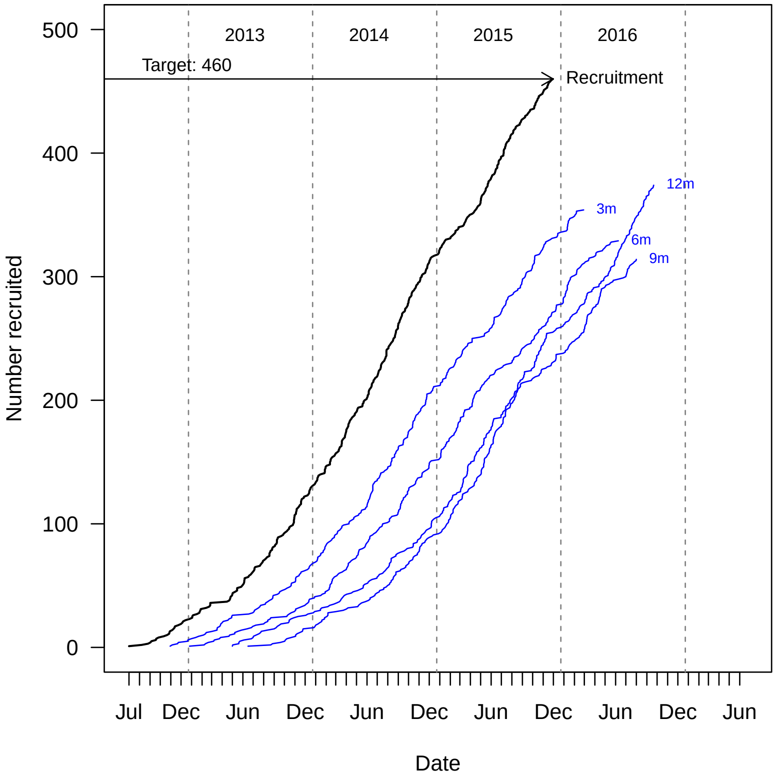

Figure 5: Progress of recruitment (—) and follow-up (—) for WOLLF

4.2 Group sequential adaptive design

The trial recorded DRI at 3m, 6m, 9m and 12m, with the primary outcome at 12m. From the WOLLF trial protocol [5], the planned sample size was $N = 412$, based on a standard deviation $\sigma_{12m} = 25$ and MCID of 8 for DRI; allowing 10% loss to follow-up, gave $N = 460$.

If recruitment had proceeded to target as originally planned, and assuming that $N0^*_{12m} = N1^*_{12m} = 206$, then the information at the trial end would be given by $I^*_{End} = 103/25^2 = 0.165$. Inspection of the planned recruitment schedules for the trial suggests that three interim analyses would have been feasible for WOLLF. Let us assume, for the purposes of planning the interim analyses, that the numbers in the intervention arms are always equal, and that interim analyses could have feasibly occurred when $N^*_{12m} = 50$, $N^*_{12m} = 100$ and $N^*_{12m} = 150$. The expected numbers of early outcomes at these three interim analyses could have been as follows, based on the patterns of follow-up that might have been expected before recruitment started:

Interim	N^*_{3m}	N^*_{6m}	N^*_{9m}	N^*_{12m}
1	120	100	70	50
2	240	200	140	100
3	360	300	210	150

Using the approach of Parsons et al.[7], we further assume that the standard deviations are the same for the early outcomes as for the final outcome $\sigma^*_{3m} = \sigma^*_{6m} = \sigma^*_{9m} = \sigma^*_{12m} = 25$, and that the correlations between outcomes are all equal such that $\rho^*_{3m,6m} = \rho^*_{3m,9m} = \rho^*_{3m,12m} = \rho^*_{6m,9m} = \rho^*_{6m,12m} = \rho^*_{9m,12m} = 0.5$. If this is the case, then given the expected standard deviations $\sigma^* = 25$ and correlations $\rho^* = 0.5$ the expected information (I^*) required at the three interim analyses are as follows, $I^*_1 = 0.025$, $I^*_2 = 0.05$ and $I^*_3 = 0.075$. Using the four selected design options for futility (cumulative) stopping probabilities, (a) $\alpha^*_l = (0.080, 0.160, 0.240, 0.975)$, (b) $\alpha^*_l = (0.160, 0.320, 0.480, 0.975)$, (c) $\alpha^*_l = (0.240, 0.480, 0.7200.975)$ and (d) $\alpha^*_l = (0.320, 0.640, 0.960, 0.975)$ and efficacy stopping probabilities (for all options) $\alpha^*_u = (0.001, 0.005, 0.010, 0.025)$, gives the following lower and upper stopping boundaries for the test

statistic (Z) at each interim analysis and the trial end:

Design		1	2	3	End		1	2	3	End
(a)	l_a	-1.41	-1.14	-0.91	2.09	u_a	3.09	2.62	2.45	2.09
(b)	l_b	-0.99	-0.59	-0.20	2.08	u_b	3.09	2.62	2.45	2.08
(c)	l_c	-0.71	-0.15	0.49	2.04	u_c	3.09	2.62	2.45	2.04
(d)	l_d	-0.47	0.29	1.75	1.36	u_d	3.09	2.62	2.45	1.36

These stopping boundaries were used to simulate the progress of the trial and assess whether the trial would have stopped at each of the interim analyses.

4.3 Results

The numbers of observed trial participants providing data at each interim analysis is shown in Table 11 and the total numbers of participants recruited at the time each interim analysis took place is shown in Table 14.

Table 11: Numbers of observed and expected participants providing 3m, 6m, 9m and 12m outcome data at each interim analysis and the trial end for WOLLF.

Interim	Observed				Expected			
	N_{3m}	N_{6m}	N_{9m}	N_{12m}	N^*_{3m}	N^*_{6m}	N^*_{9m}	N^*_{12m}
1	115	84	51	37	120	100	70	50
2	188	136	85	74	240	200	140	100
3	255	217	173	156	360	300	210	150
End	354	329	314	374	412	412	412	412

Estimated treatment effects at 12m, from the longitudinal model exploiting early outcome data (β_{12m}) and using final outcome data only ($\beta0_{12m}$), test statistics (Z and $Z0$) and information thresholds used to trigger interim analyses I^* and observed information $I = 1/\text{var}(\beta_{12m})$ are shown in Table 12.

Table 12: Means and estimates of treatment effects at each interim analysis and the trial end for WOLLF.

Interim	$m1_{12m}$	$m0_{12m}$	Δ	β_{12m}	var(β_{12m})	Z	I	I^*	$\beta0_{12m}$	var($\beta0_{12m}$)	$Z0$
1	44.67	42.88	-1.79	-0.36	39.72	-0.06	0.025	0.025	-1.79	63.67	-0.22
2	40.06	44.17	4.11	-2.76	19.59	-0.62	0.051	0.050	4.11	31.40	0.73
3	41.98	44.13	2.16	0.19	13.25	0.05	0.075	0.075	2.16	17.82	0.51
End	45.51	42.36	-3.14	-3.65	6.97	-1.38	0.143	0.165	-3.14	7.29	-1.16

$m0_{12m}$ and $m1_{12m}$ are the means for the Control and NPWT arms and $\Delta = m0_{12m} - m1_{12m}$, and $\Delta = \beta0_{12m}$

Table 13 shows the estimated correlations and standard deviations at each interim analysis and Figure 6 shows scatter plots of the observed data between time-points. The estimated correlations were all generally larger than those used in the model to determine the timings of the interim analyses (i.e. $\rho^* = 0.5$ for all pairs of times) and standard deviations were much as expected (i.e. $\sigma^* = 25$ for all times), at all interim analyses.

Test statistic Z is in the continuation region (between upper and lower boundaries) at interim analysis 1, for all boundary settings (a) to (d) (see Table 12 and boundaries in Section 4.2). However, at interim analysis 2, Z falls below the lower boundaries for settings (b) to (d), indicating that the trial would have been stopped for futility. Z does not fall below the lower boundary or above the upper boundary for setting a, so the trial would not have stopped for this setting. Looking at the overrunning analysis, gives the numbers of trial participants providing data at each interim analysis shown in Table 14 and estimated model parameters and test statistics in Table 15.

The test statistics (Z) from the overrunning analyses shown in Table 15 confirm the results of the stopping decisions based on the interim data (Table 12). In summary, the test statistics from the interim analysis in Table

Table 13: Estimates of correlations and standard deviations at each interim analysis and the trial end; the design model assumed $\rho^* = 0.5$, for all pairs of times and $\sigma^* = 25$ for all times for WOLLF.

Interim	$\rho_{3m,6m}$	$\rho_{3m,9m}$	$\rho_{3m,12m}$	$\rho_{6m,9m}$	$\rho_{6m,12m}$	$\rho_{9m,12m}$	σ_{3m}	σ_{6m}	σ_{9m}	σ_{12m}
1	0.66	0.61	0.59	0.83	0.77	0.88	22.65	23.50	26.67	25.71
2	0.62	0.61	0.58	0.78	0.78	0.89	22.08	23.94	24.75	24.72
3	0.59	0.52	0.48	0.72	0.71	0.82	21.15	23.59	25.16	26.45
End	0.65	0.55	0.58	0.67	0.73	0.74	21.25	23.92	25.59	26.22

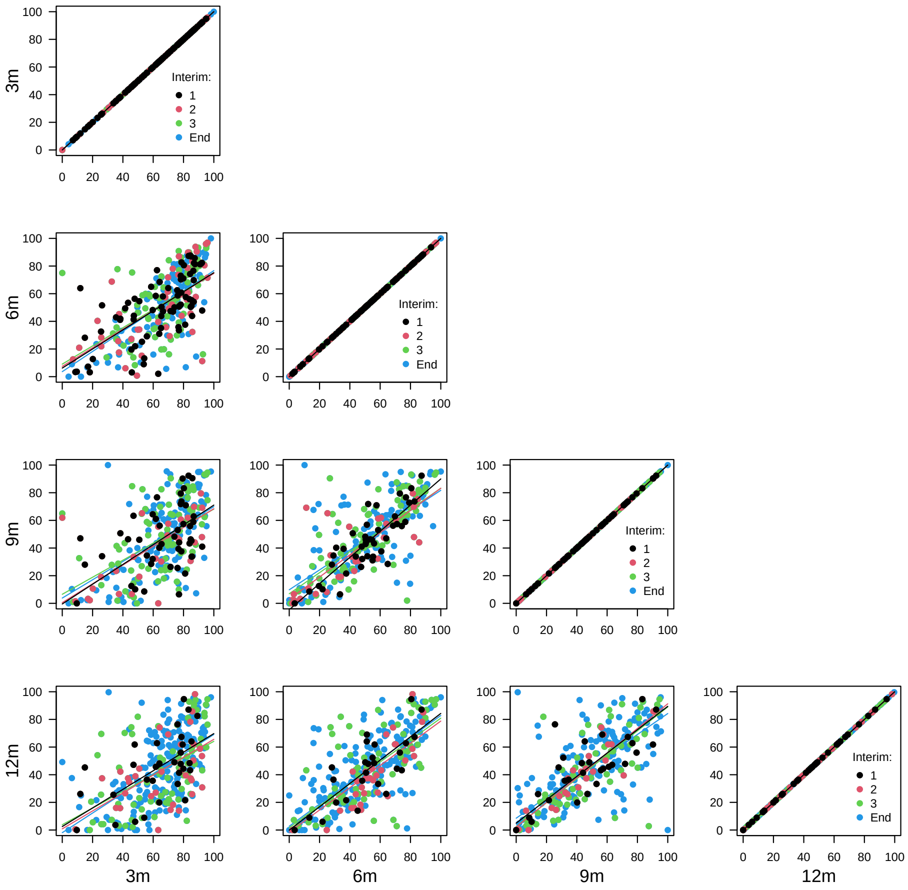

Figure 6: Scatter plots of the observed data between time-points, with fitted lines, for each interim analysis and trial end for WOLLF

Table 14: Numbers of participants providing 3m, 6m, 9m and 12m outcome data at each overrunning analysis, and the total trial recruitment (N) and trial length (in months) for WOLLF

Interim	N_{3m}	N_{6m}	N_{9m}	N_{12m}	N	Months
1	156	144	127	160	201	23
2	221	204	196	242	293	27
3	288	266	256	305	373	34
End	354	329	314	374	460	50

12, and the stopping boundaries from Section 4.2, indicate that the WOLLF RCT would have stopped for futility at the second interim analysis for design settings (b) to (d).

Table 15: Means and estimates of treatment effects at each overrunning analysis and the trial end for WOLLF.

Interim	$m1_{12m}$	$m0_{12m}$	Δ	β_{12m}	var(β_{12m})	Z	I	$\beta0_{12m}$	var($\beta0_{12m}$)	$Z0$
1	42.38	43.33	0.95	-0.84	17.35	-0.20	0.058	0.95	17.98	0.22
2	45.00	42.20	-2.80	-3.46	11.31	-1.03	0.088	-2.80	11.72	-0.82
3	44.94	43.76	-1.18	-1.91	8.91	-0.64	0.112	-1.18	9.25	-0.39
End	45.51	42.36	-3.14	-3.65	6.97	-1.38	0.143	-3.14	7.29	-1.16

$m0_{12m}$ and $m1_{12m}$ are the means for the control and NPWT arms and $\Delta = m0_{12m} - m1_{12m}$, and $\Delta = \beta0_{12m}$

5 The Full UK RCT of Arthroscopic Surgery for Hip Impingement versus best cONServative care (FASHION) trial

5.1 Details

The full UK RCT of arthroscopic surgery for hip impingement versus best conservative care trial (FASHION) was a pragmatic, multicentre, assessor-blinded randomised controlled trial, undertaken at 23 NHS hospitals in the UK [8, 9, 10]. FASHION recruited $N = 348$ adult patients with femoroacetabular impingement syndrome who presented at these hospitals, and randomly allocated them (1:1) to receive either hip arthroscopic surgery ($N1 = 171$) or personalised hip therapy ($N0 = 177$), between July 2012 and July 2016; Figure 7 shows the progress of recruitment and followed-up (FU) at 6 and 12 months for FASHION. Personalised hip therapy (PHT, control) is an individualised, supervised, and progressive physiotherapist-led programme of conservative care. The primary outcome was hip-related quality of life, as measured by the patient-reported International Hip Outcome Tool (iHOT-33) at 12 months after randomisation, with early outcome assessed at 6 months. iHOT-33 provides a 100-point score, with 100 representing no pain and perfect function, and lower scores indicating pain and poor function.
The primary result of the trial was that there was a statistically significant difference in iHOT-33 score at 12 months; the mean score in the Surgery group was 58.8 ($N1_{12m} = 158$) and in the PHT group 49.7 ($N0_{12m} = 163$), a difference of 9.1 (95%CI; 3.3 to 14.9) (see Table 2 in main clinical paper [9]). The trial took 60 months (recruitment plus follow-up) to complete.

5.2 Group sequential adaptive design

The trial recorded an early iHOT-33 outcome at 6m, with the primary outcome at 12m. From the FASHION trial protocol [8], the planned sample size was $N = 292$, based on standard deviation $\sigma_{12m} = 16$ and MCID of 6 for iHOT-33. Allowing 15% loss to FU, gave $N = 344$. However, this plan was based on the very limited information that was available when the trial started. iHOT-33 was a newly developed outcome measure, with little data available on its natural variation in the target population of the trial, other than from pilot work and baseline data [10]. Therefore, at the design stage of the original trial it was evident that the selected value for σ_{12m} was likely to be an unreliable (imprecise) estimate. It quickly became clear as the trial proceeded that $\sigma_{12m} = 16$ was far too low, with emerging data suggesting that $\sigma_{12m} = 24$ was more realistic. However, the trial sample was not changed after recruitment started for FASHION, as the new working sample size model based on $\sigma_{12m} = 24$ and MCID of 8, gave the same effect size estimate (0.38) and overall sample size as the original model. Given that the timing of the interim analyses in the adaptive design is driven in large part by σ_{12m}, via accumulated information, we chose this latter formulation to motivate our design.
If recruitment had proceeded to target as originally planned, and we assume that $N0^*_{12m} = N1^*_{12m} = 146$, then the information at the trial end would be given by $I^*_{End} = 73/24^2 = 0.127$. Inspection of the planned recruitment schedules for the trial suggests that two interim analyses would have been feasible for FASHION. Assume now, for the purposes of planning the interim analyses, that numbers in the intervention arms are always equal, and that analyses could have feasibly occurred when $N^*_{12m} = 50$ and $N^*_{12m} = 100$. The expected numbers of early outcomes at the interim analyses could have been as follows, based on the patterns of follow-up that might have been expected before recruitment started:

Interim	N^*_{6m}	N^*_{12m}
1	100	50
2	200	100

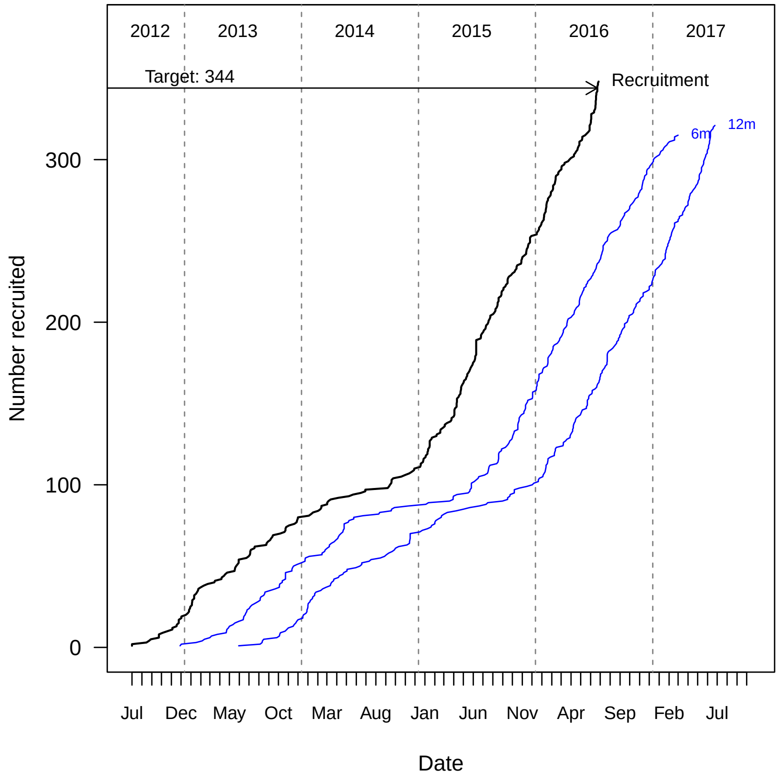

Figure 7: Progress of recruitment (—) and follow-up (—) for FASHION.

Assuming that the standard deviations are the same for the early outcomes as for the final outcome $\sigma^*_{6m} = \sigma^*_{12m} = 24$, and that the correlations between outcomes are such that $\rho^*_{6m,12m} = 0.5$, then the information required at the two interim analyses are as follows, $I^*_1 = 0.025$ and $I^*_2 = 0.050$. Using the following four options for futility (cumulative) stopping probabilities, (a) $\alpha^*_l = (0.080, 0.160, 0.975)$, (b) $\alpha^*_l = (0.160, 0.320, 0.975)$, (c) $\alpha^*_l = (0.240, 0.4800.975)$ and (d) $\alpha^*_l = (0.320, 0.640, 0.975)$ and efficacy stopping probabilities (for all options) $\alpha^*_u = (0.001, 0.010, 0.025)$, gives the following lower and upper stopping boundaries for the test statistic (Z) at each interim analysis and the trial end:

Design		1	2	End		1	2	End
(a)	l_a	-1.41	-1.14	2.10	u_a	3.09	2.34	2.10
(b)	l_b	-0.99	-0.59	2.09	u_b	3.09	2.34	2.09
(c)	l_c	-0.71	-0.15	2.08	u_c	3.09	2.34	2.08
(d)	l_d	-0.47	0.29	2.06	u_d	3.09	2.34	2.06

These stopping boundaries were used to simulate the progress of the trial and assess whether the trial would have stopped at each of the interim analyses.

5.3 Results

The numbers of observed trial participants providing data at each interim analysis is shown in Table 16 and the total numbers of participants recruited at the time each interim analysis took place is shown in Table 29.

Estimated treatment effects at 12m, from the longitudinal model exploiting early outcome data (β_{12m}) and using final outcome data only ($\beta 0_{12m}$), test statistics (Z and $Z0$) and information thresholds used to trigger interim

Table 16: Numbers of observed and expected participants providing 6m and 12m outcome data at each interim analysis and the trial end for FASHION.

Interim	Observed		Expected	
	N_{6m}	N_{12m}	N^*_{6m}	N^*_{12m}
1	86	62	100	50
2	208	141	200	100
End	315	321	292	292

analyses I^* and observed information $I = 1/\text{var}(\beta_{12m})$ are shown in Table 17.

Table 17: Means and estimates of treatment effects at each interim analysis and the trial end for FASHION.

Interim	$m1_{12m}$	$m0_{12m}$	Δ	β_{12m}	$\text{var}(\beta_{12m})$	Z	I	I^*	$\beta0_{12m}$	$\text{var}(\beta0_{12m})$	$Z0$
1	56.09	49.02	7.08	3.60	39.08	0.58	0.026	0.025	7.08	42.64	1.08
2	55.90	49.13	6.77	6.50	18.86	1.50	0.053	0.050	6.77	20.97	1.48
End	58.76	49.68	9.08	8.74	8.53	2.99	0.117	0.127	9.08	8.64	3.09

$m0_{12m}$ and $m1_{12m}$ are the means for the PHT and Surgery arms and $\Delta = m1_{12m} - m0_{12m}$, and $\Delta = \beta0_{12m}$

Table 18 shows the estimated correlations and standard deviations at each interim analysis and Figure 8 shows scatter plots of the observed data between time-points. The estimated standard deviations for the 12-month outcome are slightly larger than expected when planning the trial; σ_{12m} was expected to around 24 but was actually nearer to 26. This explains why the numbers of participants at the interim analysis were slightly larger than planned, as it took more participants to accumulate the required information, due to the larger than expected value for σ_{12m}.

Table 18: Estimates of correlations and standard deviations at each interim analysis and the trial end; the design model assumed $\rho^* = 0.5$, for all pairs of times and $\sigma^* = 20$ for all times for FASHION.

Interim	$\rho_{6m,12m}$	σ_{6m}	σ_{12m}
1	0.56	22.90	25.81
2	0.56	23.88	27.27
End	0.57	24.14	26.32

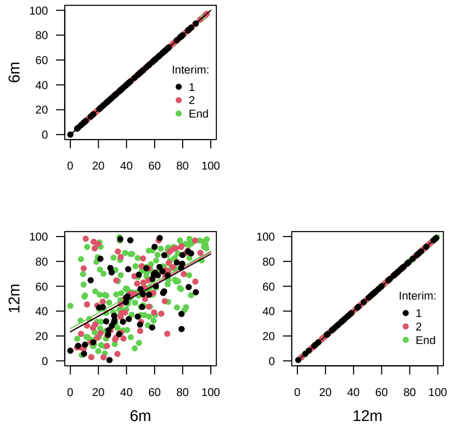

Figure 8: Scatter plots of the observed data between time-points, with fitted lines, for each interim analysis and trial end for FASHION.

Test statistic Z is in the continuation region (between upper and lower boundaries) at interim analysis 1 and 2, for all boundary settings (a) to (d).

Looking at the overrunning analysis, gives the numbers of trial participants providing data at each interim analysis shown in Table 19 and estimated model parameters and test statistics in Table 20.

Table 19: Numbers of participants providing 6m and 12m outcome data at each overrunning analysis, and the total trial recruitment (N) and trial length (in months) for FASHION.

Interim	N_{6m}	N_{12m}	N	Months
1	96	96	104	27
2	275	279	304	45
End	315	321	348	59

Table 20: Means and estimates of treatment effects at each overrunning analysis and the trial end for FASHION.

Interim	$m1_{12m}$	$m0_{12m}$	Δ	β_{12m}	var(β_{12m})	Z	I	$\beta 0_{12m}$	var($\beta 0_{12m}$)	$Z0$
1	53.44	46.96	6.48	6.52	28.95	1.21	0.035	6.48	29.31	1.20
2	58.97	49.53	9.44	9.13	9.93	2.90	0.101	9.44	10.05	2.98
End	58.76	49.68	9.08	8.74	8.53	2.99	0.117	9.08	8.64	3.09

$m0_{12m}$ and $m1_{12m}$ are the means for the PHT and Surgery arms and $\Delta = m1_{12m} - m0_{12m}$, and $\Delta = \beta 0_{12m}$

The test statistics (Z) from the overrunning analyses shown in Table 20 confirm the results of the stopping decisions based on the interim data (Table 17). In summary, the test statistics from the interim analysis in Table 17, and the stopping boundaries from Section 5.2, indicate that the FASHION RCT would not have stopped at the interim analyses for any of the design settings.

6 The Can Shoulder Arthroscopy Work (CSAW) trial

6.1 Details

The Can Shoulder Arthroscopy Work (CSAW) trial was a three-group, pragmatic, randomised (1:1:1) controlled trial that compared Arthroscopic SubAcromial Decompression (ASAD), Arthroscopy Only (AO) and Active Monitoring with Specialist Reassessment (AMSR; no surgical treatment) [11, 12]. The trial used the Oxford Shoulder Score (OSS) at 6 months (6m) after randomisation to assess participant outcomes; OSS measures a patient's experience of shoulder pain and function using 12-items scored from 0 to 4 and summed to give a score from 0 to 48 (with 0 being the worst score). OSS was also assessed at 12 months after randomisation, but no early assessment of OSS was made before the 6 months primary endpoint. The primary objective of the trial was to compare ASAD against the non-surgery ASMR arm to assess efficacy. The trial recruited $N = 210$ participants (to the ASAD and AMSR arms) from September 2012 to June 2015, randomising $N1 = 106$ to ASAD and $N0 = 104$ to AMSR; Figure 9 shows the progress of recruitment and followed-up (FU) at 6 and 12 months for CSAW.
The primary result of the trial was that there was a statistically significant difference in OSS at 6 months between ASAD and AMSR; the mean score in the ASAD group was 32.7 ($N1_{6m} = 90$) and in the AMSR group 29.4 ($N0_{6m} = 90$), a difference of 3.3 (95%CI; -0.2 to 6.8) (see Table 2 in main clinical paper [12] for adjusted difference). Although statistically significant, the difference was not considered to be of clinical importance. The trial took 48 months (recruitment plus 12 months follow-up) to complete.

6.2 Group sequential adaptive design

The trial recorded OSS at 6m and 12m, with the primary outcome at 6m. From the CSAW trial [11], planned sample size $N = 85$, based on standard deviation $\sigma_{6m} = 9$ and MCID of 4.5 for OSS, based on 90% power at the 5% level. Allowing for 15% loss to follow-up, the final trial sample size was $N = 100$ in each of the three trial intervention arms.
Unusually amongst the Trauma and Orthopaedics trials discussed here, the CSAW trial did not collect an early outcome prior to the definitive (primary) outcome at 6 months. For this reason, it was not possible to use the approach

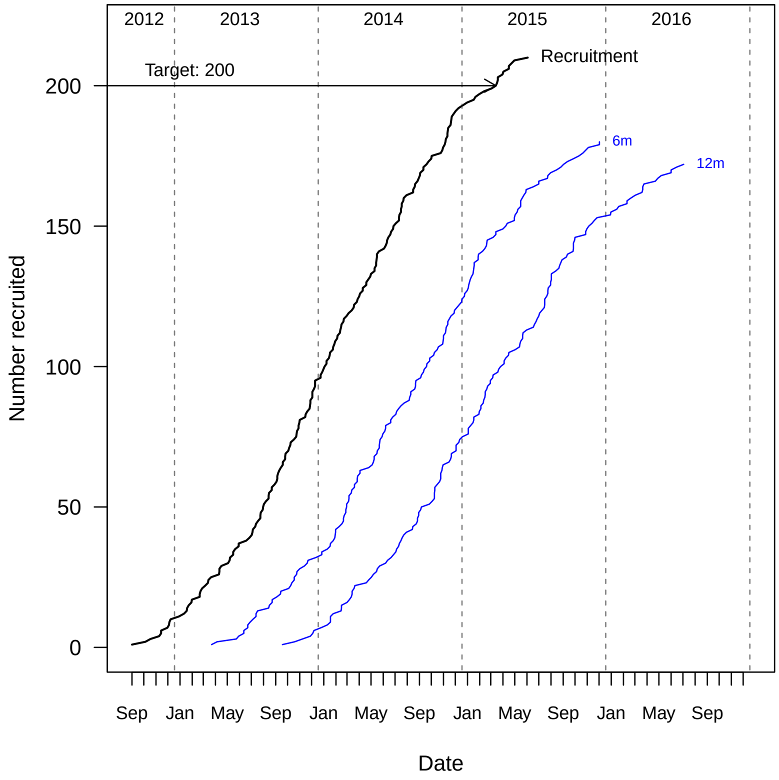

Figure 9: Progress of recruitment (—) and follow-up (—) for CSAW

of Parsons et al. [7], which required early outcome data to fully implement the methodological approach. However, in the case of no early outcome data the method of Parsons et al. reduces to the conventional group sequential setting with stopping decisions based on the 6 months primary outcome data only. In this setting, the treatment effect estimate (β_t) is given simply by the difference in 6m OSS treatment group means and sd(β_t) by the usual expression for the standard error of the difference in means.

If recruitment had proceeded to target as originally planned, and we assume that $N0^*_{6m} = N1^*_{6m} = 85$, then the information at the trial trial end would be given by $I^*_{End} = 2.5/9^2 = 0.525$. Inspection of the actual and planned recruitment schedules for the trial suggests that two interim analyses would have been feasible for CSAW. Assume now, for the purposes of planning the interim analyses, that numbers in the intervention arms are always equal, and that the interim analyses could have feasibly occurred when $N^*_{6m} = 40$ and $N^*_{6m} = 80$.

Interim	N^*_{6m}
1	40
2	80

Assuming a standard deviation $\sigma^*_{6m} = 9$, then the information required at the interim analyses are as follows, $I^*_1 = 0.123$ and $I^*_2 = 0.247$. Using the following four options for futility (cumulative) stopping probabilities, (a) $\alpha^*_l = (0.080, 0.160, 0.975)$, (b) $\alpha^*_l = (0.160, 0.320, 0.975)$, (c) $\alpha^*_l = (0.240, 0.4800.975)$ and (d) $\alpha^*_l = (0.320, 0.640, 0.975)$ and efficacy stopping probabilities (for all options) $\alpha^*_u = (0.001, 0.010, 0.025)$, gives the following lower and upper stopping boundaries for the test statistic (Z) at each interim analysis and the trial end:

Design		1	2	End		1	2	End
(a)	l_a	-1.41	-1.14	2.08	u_a	3.09	2.34	2.08
(b)	l_b	-0.99	-0.59	2.08	u_b	3.09	2.34	2.08
(c)	l_c	-0.71	-0.15	2.07	u_c	3.09	2.34	2.07
(d)	l_d	-0.47	0.29	2.06	u_d	3.09	2.34	2.06

These stopping boundaries were used to simulate the progress of the trial and assess whether the trial would have stopped at each of the interim analyses.

6.3 Results

The numbers of observed trial participants providing data at each interim analysis is shown in Table 21 and the total numbers of participants recruited at the time each interim analysis took place is shown in Table 24.

Table 21: Numbers of observed and expected participants providing 6m outcome data at each interim analysis and the trial end for CSAW.

Interim	Observed N_{6m}	Expected N^*_{6m}
1	79	40
2	137	80
End	180	170

Estimated treatment effects at 6m, from the longitudinal model (β_{6m}) and using final outcome data only ($\beta 0_{6m}$), test statistics (Z and $Z0$) and information thresholds used to trigger interim analyses I^* and observed information $I = 1/\text{var}(\beta_{6m})$ are shown in Table 22. For CSAW, as there was no early outcome data, $\beta_{6m} = \beta 0_{6m}$ and $\text{var}(\beta 0_{6m}) = \text{var}(\beta_{6m})$ at all occasions.

Table 22: Means and estimates of treatment effects at each interim analysis and the trial end for CSAW.

Interim	$m1_{6m}$	$m0_{6m}$	Δ	β_{6m}	$\text{var}(\beta_{6m})$	Z	I	I^*	$\beta 0_{6m}$	$\text{var}(\beta 0_{6m})$	$Z0$
1	30.05	31.47	1.42	1.42	7.49	0.52	0.134	0.123	1.42	7.49	0.52
2	29.56	31.72	2.17	2.17	4.01	1.08	0.249	0.247	2.17	4.01	1.08
End	29.37	32.68	3.31	3.31	3.08	1.89	0.325	0.525	3.31	3.08	1.89

$m0_{6m}$ and $m1_{6m}$ are the means for the AMSR (control) and ASAD arms and $\Delta = m1_{6m} - m0_{6m}$, and $\Delta = \beta 0_{6m}$

Table 23 shows the estimated standard deviations at each interim analysis. The estimated standard deviations for the 6-month outcome (σ_{6m}) at the interim analysis was larger than expected; σ_{6m} was expected to around 9 but was actually nearer to 12. This explains why the numbers of participants at the interim analysis were larger than planned, as it took more participants than expected to accumulate the required information.

Table 23: Estimates of standard deviation at each interim analysis and the trial end; the design model assumed $\sigma^* = 9$ for all times for CSAW.

Interim	σ_{6m}
1	12.2
2	11.7
End	11.8

Test statistic Z is in the continuation region (between upper and lower boundaries) at the interim analysis, for all boundary settings (a) to (d). Note that because we have no early outcome data for CSAW, Z is equal to $Z0$ at all analyses.
Looking at the overrunning analysis gives the numbers of trial participants providing data at each interim analysis

shown in Table 24 and estimated model parameters and test statistics in Table 25.

Table 24: Numbers of participants providing 6m outcome data at each overrunning analysis, and the total trial recruitment (N) and trial length (in months) for CSAW.

Interim	N_{6m}	N	Months
1	125	142	21
2	169	195	28
End	180	210	39

Table 25: Means and estimates of treatment effects at each overrunning analysis and the trial end for CSAW.

Interim	$m1_{6m}$	$m0_{6m}$	Δ	β_{6m}	var(β_{6m})	Z	I	$\beta0_{6m}$	var($\beta0_{6m}$)	$Z0$
1	29.95	31.68	1.73	1.73	4.40	0.82	0.227	1.73	4.40	0.82
2	29.30	33.16	3.85	3.85	3.19	2.16	0.314	3.85	3.19	2.16
End	29.37	32.68	3.31	3.31	3.08	1.89	0.325	3.31	3.08	1.89

$m0_{6m}$ and $m1_{6m}$ are the means for the AMSR (control) and ASAD arms and $\Delta = m1_{6m} - m0_{6m}$, and $\Delta = \beta0_{6m}$

The test statistics (Z) from the overrunning analysis shown in Table 25 confirm the results of the stopping decisions based on the interim data (Table 22). In summary, the test statistics from the interim analysis in Table 22, and the stopping boundaries from Section 6.2, indicate that the CSAW RCT would not have stopped at the interim analysis for any of the design settings.

7 The FIXation of Distal Tibia fractures (FIXDT) trial

7.1 Details

The Fixation of Distal Tibia Fractures (FIXDT) trial compared intramedullary nail fixation with locking plate fixation for adult patients with a displaced fracture of the distal tibia [13, 14]. FIXDT recruited 321 patients after a displaced fracture of the distal tibia between April 2013 and April 2016. The main objective of the trial was to assess the disability of patients after surgical repair with either intramedullary nail fixation (Nail, control) or locking plate fixation (Plate). The primary outcome of the trial was the Disability Rating Index (DRI) score (range, 0 = no disability to 100 = completely disabled) at 6 months (6m), with early outcome measured at 3 months (3m), with long-term outcomes also measured at 12 months. There were $N = 321$ participants who were randomised in a 1:1 ratio to either Nail ($N0 = 161$) or Plate ($N1 = 160$) fixation; Figure 10 shows the progress of recruitment and followed-up (FU) at 3, 6 and 12 months for FIXDT.
The primary result of the trial was that there was no statistically significant difference in DRI score at 6 months; the mean score in the Nail group was 29.8 ($N1_{6m} = 142$) and in the Plate group 33.8 ($N0_{6m} = 140$), a difference of -4.0 (95%CI; -9.6 to 1.6) (see Table 2 in main paper [14]). The trial took 42 months (recruitment plus follow-up) to complete (excluding the time taken for the completion of the 12 months of long-term follow-up).

7.2 Group sequential adaptive design

The trial recorded an early DRI outcome at 3m, with the primary outcome at 6m. From the FIXDT trial protocol [13], the planned sample size was $N = 264$, based on standard deviation $\sigma_{6m} = 20$ and MCID of 8 for DRI (Disability rating Index; 0-100). Allowing 20% loss to FU, gave $N = 320$.
If recruitment had proceeded to target as originally planned, and we assume that $N0^*_{6m} = N1^*_{6m} = 132$, then the information at the trial end would be given by $I^*_{End} = 66/20^2 = 0.165$. Inspection of the planned recruitment schedules for the trial suggests that two interim analyses would have been feasible for FIXDT. Assume now, for the purposes of planning the interim analyses, that numbers in the intervention arms are always equal, and that analyses could have feasibly occurred when $N^*_{6m} = 50$ and $N^*_{6m} = 100$. The expected numbers of early outcomes at the interim analyses could have been as follows, based on the patterns of follow-up that might have been expected

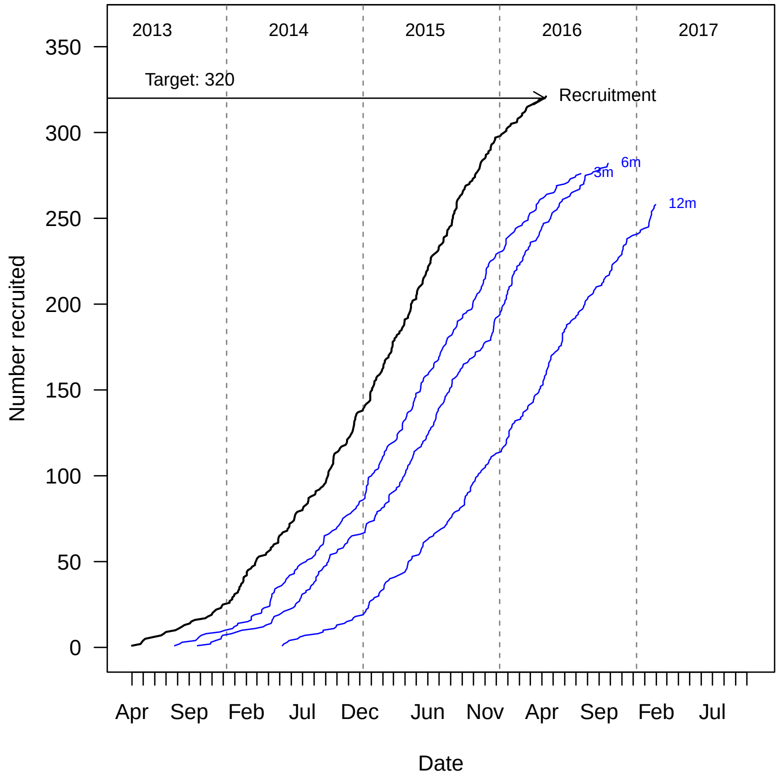

Figure 10: Progress of recruitment (—) and follow-up (—) for FIXDT

before recruitment started:

Interim	N^*_{3m}	N^*_{6m}
1	100	50
2	200	100

Assuming that the standard deviations are the same for the early outcomes as for the final outcome $\sigma^*_{3m} = \sigma^*_{6m} = 20$, and that the correlations between outcomes are such that $\rho^*_{3m,6m} = 0.5$, then the information required at the two interim analyses are as follows, $I^*_1 = 0.036$ and $I^*_2 = 0.071$. Using the following four options for futility (cumulative) stopping probabilities, (a) $\alpha^*_l = (0.080, 0.160, 0.975)$, (b) $\alpha^*_l = (0.160, 0.320, 0.975)$, (c) $\alpha^*_l = (0.240, 0.4800.975)$ and (d) $\alpha^*_l = (0.320, 0.640, 0.975)$ and efficacy stopping probabilities (for all options) $\alpha^*_u = (0.001, 0.010, 0.025)$, gives the following lower and upper stopping boundaries for the test statistic (Z) at each interim analysis and the trial end:

Design		1	2	End		1	2	End
(a)	l_a	-1.41	-1.14	2.09	u_a	3.09	2.34	2.09
(b)	l_b	-0.99	-0.59	2.09	u_b	3.09	2.34	2.09
(c)	l_c	-0.71	-0.15	2.08	u_c	3.09	2.34	2.08
(d)	l_d	-0.47	0.29	2.06	u_d	3.09	2.34	2.06

These stopping boundaries were used to simulate the progress of the trial and assess whether the trial would have stopped at each of the interim analyses.

7.3 Results

The numbers of observed trial participants providing data at each interim analysis is shown in Table 26 and the total numbers of participants recruited at the time each interim analysis took place is shown in Table 29.

Table 26: Numbers of observed and expected participants providing 3m and 6m outcome data at each interim analysis and the trial end for FIXDT.

Interim	Observed		Expected	
	N_{3m}	N_{6m}	N^*_{3m}	N^*_{6m}
1	105	79	100	50
2	178	146	200	100
End	273	282	264	264

Estimated treatment effects at 6m, from the longitudinal model exploiting early outcome data (β_{6m}) and using final outcome data only ($\beta 0_{6m}$), test statistics (Z and $Z0$) and information thresholds used to trigger interim analyses I^* and observed information $I = 1/\text{var}(\beta_{6m})$ are shown in Table 27.

Table 27: Means and estimates of treatment effects at each interim analysis and the trial end for FIXDT.

Interim	$m1_{6m}$	$m0_{6m}$	Δ	β_{6m}	var(β_{6m})	Z	I	I^*	$\beta 0_{6m}$	var($\beta 0_{6m}$)	$Z0$
1	30.02	32.32	2.29	1.14	27.06	0.22	0.037	0.036	2.29	30.77	0.41
2	32.44	31.92	-0.52	-2.85	13.86	-0.76	0.072	0.071	-0.52	15.14	-0.13
End	33.80	29.84	-3.96	-4.27	8.03	-1.51	0.125	0.165	-3.96	8.08	-1.39

$m0_{6m}$ and $m1_{6m}$ are the means for the Nail and Plate arms and $\Delta = m0_{6m} - m1_{6m}$, and $\Delta = \beta 0_{6m}$

Table 28 shows the estimated correlations and standard deviations at each interim analysis and Figure 11 shows scatter plots of the observed data between time-points. The estimated standard deviations for the 6-month outcome are much larger than expected when planning the trial; σ_{6m} was expected to around 20 but was actually nearer to 24. This explains why the numbers of participants at the interim analysis were larger than planned, as it took more participants to accumulate the required information, due to the larger than expected value for σ_{6m}.

Table 28: Estimates of correlations and standard deviations at each interim analysis and the trial end; the design model assumed $\rho^* = 0.5$, for all pairs of times and $\sigma^* = 20$ for all times for FIXDT.

Interim	$\rho_{3m,6m}$	σ_{3m}	σ_{6m}
1	0.61	20.13	24.56
2	0.65	20.06	23.70
End	0.65	20.01	24.07

Test statistic Z is in the continuation region (between upper and lower boundaries) at interim analysis 1, for all boundary settings (a) to (d). However, at interim analysis 2, Z falls below the lower boundaries for settings (b) to (d), indicating that the trial would have been stopped for futility.
Looking at the overrunning analysis, gives the numbers of trial participants providing data at each interim analysis shown in Table 29 and estimated model parameters and test statistics in Table 30.

Table 29: Numbers of participants providing 3m and 6m outcome data at each overrunning analysis, and the total trial recruitment (N) and trial length (in months) for FIXDT.

Interim	N_{3m}	N_{6m}	N	Months
1	140	148	159	21
2	210	217	243	27
End	273	282	321	42

The test statistics (Z) from the overrunning analysis shown in Table 30 confirm the results of the stopping decisions based on the interim data (Table 27). In summary, the test statistics from the interim analysis in Table 27,

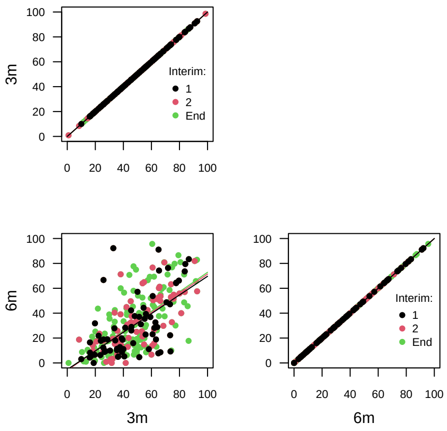

Figure 11: Scatter plots of the observed data between time-points, with fitted lines, for each interim analysis and trial end for FIXDT.

Table 30: Means and estimates of treatment effects at each overrunning analysis and the trial end for FIXDT.

Interim	$m1_{6m}$	$m0_{6m}$	Δ	β_{6m}	var(β_{6m})	Z	I	$\beta0_{6m}$	var($\beta0_{6m}$)	$Z0$
1	32.81	31.12	-1.68	-2.58	15.28	-0.66	0.065	-1.68	15.50	-0.43
2	35.36	29.68	-5.68	-6.22	10.59	-1.91	0.094	-5.68	10.69	-1.74
End	33.80	29.84	-3.96	-4.27	8.03	-1.51	0.125	-3.96	8.08	-1.39

$m0_{6m}$ and $m1_{6m}$ are the means for the Nail and Plate arms and $\Delta = m0_{6m} - m1_{6m}$, and $\Delta = \beta0_{6m}$

and the stopping boundaries from Section 7.2, indicate that the FIXDT RCT would have stopped for futility at the second interim analysis for design settings (b) to (d).

8 The Total Or Partial Knee Arthroplasty Trial (TOPKAT)

8.1 Details

The Total Or Partial Knee Arthroplasty Trial (TOPKAT) trial compared total knee replacement (TKR) to partial knee replacement (PKR) for patients with medial compartment osteoarthritis of the knee [15, 16]. The trial used the Oxford Knee Score (OKS) at 5 years (5y) after randomisation to assess participant outcomes; OKS measures a patient's experience of knee pain and function using 12-items scored from 0 to 4 and summed to give a score from 0 to 48 (with 0 being the worst score). The trial recruited $N = 528$ participants from January 2010 to September 2013, randomising $N0 = 264$ to TKR and $N1 = 264$ to PKR, on a 1:1 basis. Early outcomes were assessed on a yearly basis at 1, 2, 3 and 4 years; Figure 12 shows the progress of recruitment and followed-up (FU) at 2 months, 1, 2,3 4 and 5 years for TOPKAT.
The primary result of the trial was that there was no statistically significant difference in OKS at 5 years; the mean score in the TKR group was 37.0 ($N0_{5y} = 231$) and in the PKR group 38.0 ($N0_{5y} = 233$), a difference of 1.0 (95%CI; -0.4 to 2.5) (see Table 2 in main clinical paper [16]). The trial took 96 months (recruitment plus follow-up) to complete.

8.2 Group sequential adaptive design

The trial recorded early OKS outcome at 2m, 1y, 2y, 3y and 4y, with the primary outcome at 5y. From the TOPKAT trial [15], planned sample size $N = 500$, based on standard deviation $\sigma_{5y} = 8$ and MCID of 2 for OKS.
Recruitment to the trial was rapid (just over 3 and a half years), relative to the definitive trial endpoint at 5 years FU. Therefore, the window of opportunity, between some 5y final outcome data being available and recruitment completion was non-existent. That is, no 5y outcome data were available, prior to recruitment completing and as such the methodology we are investigating here, assessing possible early stopping of the trial, could not have been

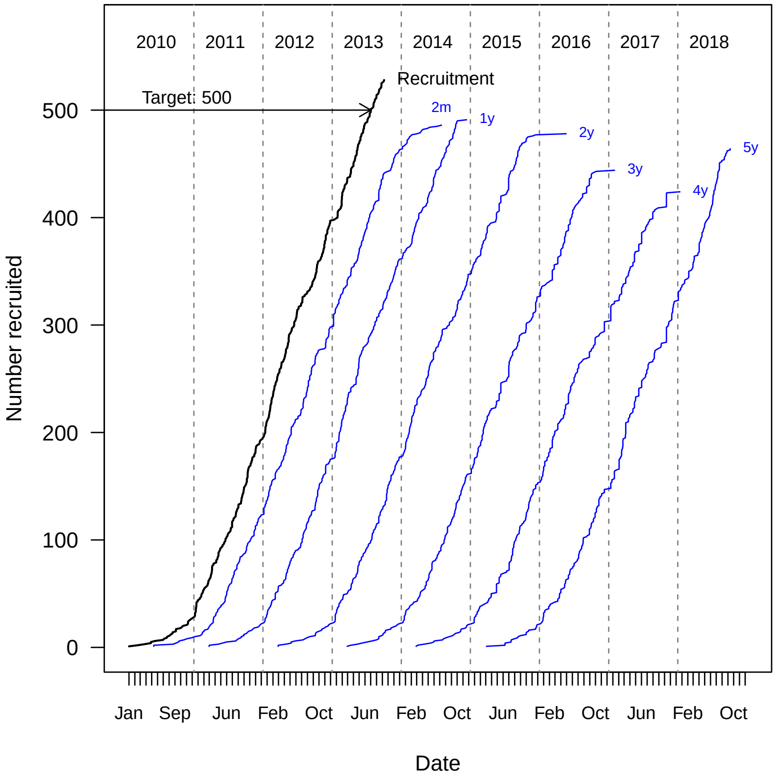

Figure 12: Progress of recruitment (—) and follow-up (—) for TOPKAT

used.

8.3 Results

As Figure 12 shows the window of opportunity, between some 5y final outcome data being available and recruitment completion was non-existent for TOPKAT and as such the methodology we are investigating here, assessing possible early stopping of the trial, could not have been used. Therefore, the modelling approach used for the other RCTs could not have been used for TOPKAT.

Competing interests

All authors have previously received or are currently in receipt of funding from the National Institute of Health Research (NIHR).

References

[1] Achten, J., Parsons, N.R., Edlin, R.P., Griffin, D.R., Costa, M.L.: A randomised controlled trial of total hip arthroplasty versus resurfacing arthroplasty in the treatment of young patients with arthritis of the hip joint. BMC Musculoskeletal Disorders **11**, 8 (2010)

[2] Costa, M.L., Achten, J., Parsons, N.R., Edlin, R.P., Foguet, P., Prakash, U., Griffin, D.R., Young Adult Hip Arthroplasty, T.: Total hip arthroplasty versus resurfacing arthroplasty in the treatment of patients with arthritis of the hip joint: single centre, parallel group, assessor blinded, randomised controlled trial. BMJ **344**, 2147 (2012)

[3] Costa, M.L., Achten, J., Parsons, N.R., Rangan, A., Edlin, R.P., Brown, J., Lamb, S.E.: UK DRAFFT - a randomised controlled trial of percutaneous fixation with kirschner wires versus volar locking-plate fixation in the treatment of adult patients with a dorsally displaced fracture of the distal radius. BMC Musculoskeletal Disorders **12**, 201 (2011)

[4] Costa, M.L., Achten, J., Parsons, N.R., Rangan, A., Griffin, D., Tubeuf, S., Lamb, S.E., Group, D.S.: Percutaneous fixation with kirschner wires versus volar locking plate fixation in adults with dorsally displaced fracture of distal radius: randomised controlled trial. BMJ **349**, 4807 (2014)

[5] Achten, J., Parsons, N.R., Bruce, J., Petrou, S., Tutton, E., Willett, K., Lamb, S.E., Costa, M.L.: Protocol for a randomised controlled trial of standard wound management versus negative pressure wound therapy in the treatment of adult patients with an open fracture of the lower limb: UK wound management of lower limb fractures (UK WOLLF). BMJ Open **5**(9), 009087 (2015)

[6] Costa, M.L., Achten, J., Bruce, J., Tutton, E., Petrou, S., Lamb, S.E., Parsons, N.R., Collaboration, U.W.: Effect of negative pressure wound therapy vs standard wound management on 12-month disability among adults with severe open fracture of the lower limb: The WOLLF randomized clinical trial. JAMA **319**(22), 2280–2288 (2018)

[7] Parsons, N., Stallard, N., Parsons, H., Wells, P., Underwood, M., Mason, J., Metcalfe, A.: An adaptive two-arm clinical trial using early endpoints to inform decision making: design for a study of sub-acromial spacers for repair of rotator cuff tendon tears. Trials **20**(1), 694 (2019)

[8] Griffin, D.R., Dickenson, E.J., Wall, P.D., Donovan, J.L., Foster, N.E., Hutchinson, C.E., Parsons, N., Petrou, S., Realpe, A., Achten, J., Achana, F., Adams, A., Costa, M.L., Griffin, J., Hobson, R., Smith, J., Group, F.A.S.: Protocol for a multicentre, parallel-arm, 12-month, randomised, controlled trial of arthroscopic surgery versus conservative care for femoroacetabular impingement syndrome (FASHION). BMJ Open **6**(8), 012453 (2016)

[9] Griffin, D.R., Dickenson, E.J., Wall, P.D.H., Achana, F., Donovan, J.L., Griffin, J., Hobson, R., Hutchinson, C.E., Jepson, M., Parsons, N.R., Petrou, S., Realpe, A., Smith, J., Foster, N.E., Group, F.A.S.: Hip arthroscopy versus best conservative care for the treatment of femoroacetabular impingement syndrome (UK FASHION): a multicentre randomised controlled trial. Lancet **391**(10136), 2225–2235 (2018)

[10] Griffin, D.R., Dickenson, E.J., Wall, P.D.H., Realpe, A., Adams, A., Parsons, N., Hobson, R., Achten, J., Costa, M.L., Foster, N.E., Hutchinson, C.E., Petrou, S., Donovan, J.L.: The feasibility of conducting a randomised controlled trial comparing arthroscopic hip surgery to conservative care for patients with femoroacetabular impingement syndrome: the FASHION feasibility study. Journal of Hip Preservation Surgery **3**(4), 304–311 (2016)

[11] Beard, D., Rees, J., Rombach, I., Cooper, C., Cook, J., Merritt, N., Gray, A., Gwilym, S., Judge, A., Savulescu, J., Moser, J., Donovan, J., Jepson, M., Wilson, C., Tracey, I., Wartolowska, K., Dean, B., Carr, A., Group, C.S.: The CSAW study (can shoulder arthroscopy work?) - a placebo-controlled surgical intervention trial assessing the clinical and cost effectiveness of arthroscopic subacromial decompression for shoulder pain: study protocol for a randomised controlled trial. Trials **16**, 210 (2015)

[12] Beard, D.J., Rees, J.L., Cook, J.A., Rombach, I., Cooper, C., Merritt, N., Shirkey, B.A., Donovan, J.L., Gwilym, S., Savulescu, J., Moser, J., Gray, A., Jepson, M., Tracey, I., Judge, A., Wartolowska, K., Carr, A.J., Group, C.S.: Arthroscopic subacromial decompression for subacromial shoulder pain (CSAW): a multicentre, pragmatic, parallel group, placebo-controlled, three-group, randomised surgical trial. Lancet **391**(10118), 329–338 (2018)

[13] Achten, J., Parsons, N.R., McGuinness, K.R., Petrou, S., Lamb, S.E., Costa, M.L.: UK fixation of distal tibia fractures (UK FIXDT): protocol for a randomised controlled trial of 'locking' plate fixation versus intramedullary nail fixation in the treatment of adult patients with a displaced fracture of the distal tibia. BMJ Open **5**(9), 009162 (2015)

[14] Costa, M.L., Achten, J., Griffin, J., Petrou, S., Pallister, I., Lamb, S.E., Parsons, N.R., Fix, D.T.T.I.: Effect of locking plate fixation vs intramedullary nail fixation on 6-month disability among adults with displaced fracture of the distal tibia: The UK FIXDT randomized clinical trial. JAMA **318**(18), 1767–1776 (2017)

[15] Beard, D., Price, A., Cook, J., Fitzpatrick, R., Carr, A., Campbell, M., Doll, H., Campbell, H., Arden, N., Cooper, C., Davies, L., Murray, D.: Total or partial knee arthroplasty trial - TOPKAT: study protocol for a randomised controlled trial. Trials **14**, 292 (2013)

[16] Beard, D.J., Davies, L.J., Cook, J.A., MacLennan, G., Price, A., Kent, S., Hudson, J., Carr, A., Leal, J., Campbell, H., Fitzpatrick, R., Arden, N., Murray, D., Campbell, M.K., Group, T.S.: The clinical and cost-effectiveness of total versus partial knee replacement in patients with medial compartment osteoarthritis (TOPKAT): 5-year outcomes of a randomised controlled trial. Lancet **394**(10200), 746–756 (2019)
