# Supplementary material for: Group sequential designs in pragmatic trials: feasibility and assessment of utility using data from a number of recent surgical RCTs
Source: BMC Med Res Methodol. 2022 Oct 1;22:256. doi: 10.1186/s12874-022-01734-2 (PMC9526271; doi:10.1186/s12874-022-01734-2)
Supplement: Supplementary file 2 — Additional file 2: Group sequential designs for longitudinal outcomes. [file 12874_2022_1734_MOESM2_ESM.pdf]

Group sequential designs for longitudinal outcomes

Nick R Parsons[1], Nigel Stallard[1], Helen Parsons[2], Aminul Haque[2], Martin Underwood[2,3], James Mason[2], Iftekhar Khan[2], Matthew L Costa[4], Damian R Griffin[2], James Griffin[2], David J Beard[4], Jonathan A Cook[5], Loretta Davies[4], Jemma Hudson[6], and Andrew Metcalfe[2,3]

[1]Statistics and Epidemiology Unit, Warwick Medical School, University of Warwick, CV4 7AL, Coventry, UK
[2]Warwick Clinical Trials Unit (WCTU), Warwick Medical School, University of Warwick, CV4 7AL, Coventry, UK
[3]University Hospital Coventry and Warwickshire (UHCW), CV2 2DX, Coventry, UK
[4]Nuffield Department of Orthopaedics, Rheumatology and Musculoskeletal Sciences (NDORMS), University of Oxford, OX3 7LD, Oxford, UK
[5]Centre for Statistics in Medicine, Nuffield Department of Orthopaedics, Rheumatology and Musculoskeletal Sciences (NDORMS), University of Oxford, OX3 7LD, Oxford, UK
[6]Health Services Research Unit (HSRU), University of Aberdeen, AB25 2ZD, Aberdeen, UK

September 27, 2022

1 Longitudinal outcomes

In a two-arm controlled clinical trial where participants are followed-up and outcomes reported at a series of t time-points, let y_{ijs} be the outcome for the ith of N participants ($i = 1, \ldots, N$), at time-point s ($s = 1, \ldots, t$) recruited into intervention arm j (0 = control and 1 = treatment) of the trial. Let us assume that the total number of participants supplying (follow-up) data at time-point s is $N0_s + N1_s$, where $N0_s$ is the number in the control arm and $N1_s$ is the number in the treatment arm. Before recruitment into the trial is completed, we assume that the number of outcome data are structured such that $N0_1 \geq N0_2 \geq \cdots \geq N0_{t-1} \geq N0_t$ and $N1_1 \geq N1_2 \geq \cdots \geq N1_{t-1} \geq N1_t$; i.e. there are always more or equivalent data available for the early than the later time-points. At the completion of the study (i.e. the end of follow-up), we also further assume that data are independent (between participants) and that the distribution of outcomes $(y_{ij1}, \ldots, y_{ijt})$ is multivariate normal, with mean $(\mu_{j1}, \ldots, \mu_{jt})$ and covariance matrix

$$\Sigma = \begin{pmatrix} \sigma_1^2 & \sigma_1\sigma_2\rho_{12} & \cdots & \sigma_1\sigma_t\rho_{1t} \\ \sigma_2\sigma_1\rho_{21} & \sigma_2^2 & \cdots & \sigma_2\sigma_t\rho_{2t} \\ \vdots & \vdots & \ddots & \vdots \\ \sigma_t\sigma_1\rho_{t1} & \sigma_t\sigma_2\rho_{t2} & \cdots & \sigma_t^2 \end{pmatrix}, \quad (1)$$

where σ_s is the standard deviation of the outcome at time s and $\rho_{ss'}$ is the correlation between endpoints at time-points s and s'.

The primary interest of the clinical trial is to estimate the effect of the treatment on the study outcome at time-point t (the primary study endpoint), which we call β_t. In order to undertake the study in the most efficient manner possible, we aim to assess the treatment effect at interim analyses (early looks) during follow-up with the possibility of stopping the study for either futility or efficacy. A number of authors have investigated this problem [1, 2, 3] and more generally group-sequential analysis for longitudinal data [4, 5]. The method of analysis proposed by Engel and Walstra [2] was formulated for a long-term and a single short-term endpoint in the setting of a fixed sample size experiment, rather than a clinical trial, and recognised the fact that information on the long-term endpoint was available from the short-term endpoint, due to the correlation between data from individual study participants at the two time points. The motivation for this approach, referred to as double-regression, was to use observations from the early outcome, which are considered to be generally less expensive, to inform inferences on more expensive definitive (final) outcomes. In the setting of a clinical trial, the cost of the longer term outcome (relatively to

the shorter outcome) is both in the resources required for longer term follow-up and the wider societal costs of potentially exposing patients to ineffective treatments, if the study ultimately provides little support for the efficacy of the intervention under test. A more general approach in the setting of a sequential trial, with a number of interim analyses, with a long-term and potentially many short-term endpoints involving comparison of an experimental arm with a control arm was first suggested by Galbraith and Marschner [3].

2 Model

Formulating as a linear longitudinal model with correlated errors, under the assumption of multivariate normality (MVN) for outcome y_i, we can write,

$$y_i \sim \mathrm{MVN}(X_i\beta, \Sigma_i), \tag{2}$$

where $\Sigma_i(\sigma, \rho)$ is the $k \times k$ covariance matrix of y_i characterised by parameters σ $(\sigma_1, \ldots, \sigma_k)$ and ρ $(\rho_{12}, \ldots, \rho_{ss'})$, X_i is a $k \times 2t$ design matrix (where study participant i has k endpoints; $1 \leq k \leq t$) and β is a $2t \times 1$ vector of unknown model parameters. β can be structured, for convenience, such that $\beta = (\beta_{10}, \beta_{20}, \ldots, \beta_{t0}, \beta_1, \beta_2, \ldots, \beta_t)$, where β_{s0} estimates the outcome mean in the control arm of the study, and β_s estimates the effect of the treatment arm relative to the control arm at time-point s. Therefore, β_t is the effect of the treatment on the study outcome at time-point t (the primary endpoint).

The maximum likelihood estimator for β, under the multivariate normal assumption for known Σ_i, is the generalized least squares estimator [6]

$$\tilde{\beta} = \left(\sum_{i=1}^{N} X_i' \Sigma_i^{-1}(\sigma, \rho) X_i\right)^{-1} \left(\sum_{i=1}^{N} X_i' \Sigma_i^{-1}(\sigma, \rho) y_i\right), \tag{3}$$

with variance given by

$$\mathrm{var}(\tilde{\beta}) = \left(\sum_{i=1}^{N} X_i' \Sigma_i^{-1}(\sigma, \rho) X_i\right)^{-1}. \tag{4}$$

Estimates $\tilde{\beta}$ and $\mathrm{var}(\tilde{\beta})$ follow naturally given covariance matrix Σ_i, which may be obtained from estimates of the correlations ρ, and standard deviations σ. These could in principle be obtained in a number of ways or could be fixed to known or expected values. Galbraith and Marschner [3] suggest using conventional mixed-effects models for analysis of correlated data to estimate the covariance parameters. This can easily be implemented, for example, by fitting separate fixed-effects for each follow-up time with an unstructured error covariance using the function `lme` in R [7] package `nlme`. Alternatively, for the purposes of monitoring information accumulation during a trial it is often beneficial to directly model the covariance structure amongst repeated outcomes rather than including random-effects to account for within individual dependences. This can be achieved using the generalized least squares model (e.g. `gls` function in `nlme`), which unlike the mixed-effects model, provides explicit estimates of the covariance parameters [8]; see example code in Section 6 for details of how models can be fitted to data in R. Either model formulation provides consistent and unbiased estimates of model parameters (see the simulation study of Section 7), under an assumed multivariate normal distribution with a general covariance structure, common follow-up times for each individual and missing outcomes that are assumed to be a consequence of the shortened follow-up duration.

Simple expressions for estimators $\tilde{\beta}_t$ and $\mathrm{var}(\tilde{\beta}_t)$ can be obtained directly for one ($t = 2$) and two ($t = 3$) early outcomes, after some algebraic manipulation, from expressions (3) and (4), if we assume that numbers in the control and intervention arms are equal ($N0_s = N1_s = N_s$), and by noting that $N_1 \geq N_2 \geq \cdots \geq N_{t-1} \geq N_t$, where the determinants of the correlation matrices

$$R_2 = \begin{pmatrix} 1 & \rho_{12} \\ \rho_{12} & 1 \end{pmatrix} \quad \text{and} \quad R_3 = \begin{pmatrix} 1 & \rho_{12} & \rho_{13} \\ \rho_{12} & 1 & \rho_{23} \\ \rho_{13} & \rho_{23} & 1 \end{pmatrix}$$

are given by $|R_2| = 1 - \rho_{12}^2$ and $|R_2| = 1 - \rho_{12}^2 - \rho_{13}^2 - \rho_{23}^2 + 2\rho_{12}\rho_{13}\rho_{23}$. The estimators $\mathrm{var}(\tilde{\beta}_2)$ and $\tilde{\beta}_2$ for one early outcome are

$$\mathrm{var}(\tilde{\beta}_2) = \frac{2\sigma_2^2}{N_2}\left(\frac{N_2 + (N_1 - N_2)|R_2|}{N_1}\right) \tag{5}$$

and

$$\tilde{\beta}_2 = \frac{1}{N_2}\sum_{i=1}^{N_2} y_{i12} - y_{i02} + \frac{\sigma_2\rho_{12}}{\sigma_1}\left(\frac{1}{N_1}\sum_{i=1}^{N_1} y_{i11} - y_{i01} - \frac{1}{N_2}\sum_{i=1}^{N_2} y_{i11} - y_{i01}\right) \tag{6}$$

and $\text{var}(\tilde{\beta}_3)$ and $\tilde{\beta}_3$ for two early outcomes are

$$\text{var}(\tilde{\beta}_3) = \frac{2\sigma_3^2}{N_3}\left(\frac{|R_3|}{|R_2|} + \frac{N_3}{N_2}\left(1 - \frac{|R_3|}{|R_2|}\right) - \frac{N_3(N_1 - N_2)\rho_{13}^2}{N_1 N_2}\right) \tag{7}$$

and

$$\begin{aligned}\tilde{\beta}_3 = \frac{1}{N_3}\sum_{i=1}^{N_3} y_{i13} - y_{i03} + \frac{\sigma_3}{N_3\sigma_2}\left(\frac{(\rho_{12}\rho_{13} - \rho_{23})}{|R_2|}\sum_{i=1}^{N_3} y_{i12} - y_{i02} - \frac{N_3(\rho_{12}\rho_{13} - \rho_{23})}{|R_2|N_2}\sum_{i=1}^{N_2} y_{i12} - y_{i02}\right) + \\ \frac{\sigma_3}{N_3\sigma_1}\left(\frac{(\rho_{12}\rho_{23} - \rho_{13})}{|R_2|}\sum_{i=1}^{N_3} y_{i11} - y_{i01} + \frac{N_3\rho_{12}(\rho_{12}\rho_{13} - \rho_{23})}{|R_2|N_2}\sum_{i=1}^{N_2} y_{i11} - y_{i01} + \frac{N_3\rho_{13}}{N_1}\sum_{i=1}^{N_1} y_{i11} - y_{i01}\right).\end{aligned} \tag{8}$$

3 Information

In order to understand how information on β_t is accumulated as a trial proceeds, the covariance matrix can be written as Σ_k to indicate that, for any study participant, it is formed from the $k \times k$ sub-matrix consisting of rows (r) and columns (c) 1 to k of the complete covariance matrix Σ. The variance matrix (4) can then be expressed as the block matrix

$$\text{var}(\tilde{\beta}) = \begin{pmatrix} \mathrm{V}_0 + \mathrm{V}_1 & \mathrm{V}_1 \\ \mathrm{V}_1 & \mathrm{V}_1 \end{pmatrix}^{-1} = \begin{pmatrix} \mathrm{V}_0^{-1} & \mathrm{V}_0^{-1} \\ \mathrm{V}_0^{-1} & \mathrm{V}_0^{-1} + \mathrm{V}_1^{-1} \end{pmatrix} \tag{9}$$

where V_0 and V_1 are $t \times t$ matrices with elements given by

$$\mathrm{V}_j[r, c] = \sum_{k=max(r,c)}^{t} (Nj_k - Nj_{k+1})\Sigma_k^{-1}[r, c] \tag{10}$$

where j is the indicator for the treatment group allocation (0 = control and 1 = treatment), $N0_{t+1} = N1_{t+1} = 0$ and $1 \le r, c \le t$. Letting $\mathrm{V} = \mathrm{V}_0^{-1} + \mathrm{V}_1^{-1}$ gives $\text{var}(\beta_t) = \mathrm{V}[t, t]$, with information on the treatment effect for the study outcome at time-point t given by $I = 1/\text{var}(\beta_t)$. The information depends on estimates of covariance parameters $\rho = \rho_{12}, \ldots, \rho_{ss'}$ and $\sigma = \sigma_1, \ldots, \sigma_k$, through Σ_k, and the number of participants ($N0_k$ and $N1_k$) with data at each time-point k. Therefore, it is possible to monitor a study as it proceeds by estimating the covariance parameters and calculating the accumulated information at any point during recruitment. Pre-set information thresholds can be used to trigger interim analyses, with stopping decisions being made based on estimates of β_t and $\text{var}(\beta_t)$ from expressions (3) and (4).

4 Implementation for a two-arm trial

For a two-arm study, with outcomes observed at t time-points, study participants are randomized to either the control or active intervention arms, and data collection proceeds until the first interim analysis when $N_{t1}, \ldots, N_{11}$, data are available; for notational convenience hereafter we let $N_{tw}, \ldots, N_{1w}$ be the number of study participants with data available at interim analysis w, where $N_{kw} = N0_{kw} + N1_{kw}$ and $1 \le k \le t$. The planned number of interim analyses W, and the criteria for triggering each analysis, are stated explicitly at the start of the study. If information accrual is used as the criterion for triggering an interim analysis, then the thresholds are stated in advance and are such that $I_1 < I_2 < \cdots < I_W$. Other criteria are perfectly possible, but these are not discussed in detail here for purposes of brevity; for instance one might decide that the minimum number of participants with outcomes at time t, N_t, must exceed some pre-set threshold. These should also similarly be stated explicitly in advance. The first interim analysis is triggered when the estimated information on the treatment effect at time t, determined from current estimates of σ and ρ, is equal to or greater than I_1. Expressions expressions (3) and (4) are then used to obtain estimates β_{t1} and $\text{var}(\beta_{t1})$, using only data available at the first look, from which the test statistic $Z_1 = \beta_{t1}/\text{sd}(\beta_{t1})$ is estimated. The observed test statistic Z_1 is then compared to pre-defined lower and

upper stopping boundaries l_1 and u_1, which are determined by the pre-set expected information I_1 at the first look and the planned error spend (see Section 5), and either the trial is stopped, for futility or efficacy, or it continues to the next interim analysis. At each subsequent interim analysis, the test statistic $Z_w = \beta_{tw}/\text{sd}(\beta_{tw})$ is calculated in an analogous manner as at the first analysis, using all available data, and compared to stopping boundaries l_w and u_w that determine whether the study is stopped early (e.g. see Parsons et al. [1]). Bias corrected point estimates of the intervention effect and associated confidence intervals can calculated, if required, using appropriate statistical methods that adjust for the interim analyses (if stopping occurred after the first analysis) [9, 10]. In general, if a study is stopped at an interim analysis, then data collection will continue for all participants recruited up to that point and these final (complete follow-up) data can also be used for making inferences in an overrunning analysis [11].

5 Sequential stopping boundaries

In a sequential trial, with a series of W interim analyses (looks), we aim to compare the two study intervention arms in order to make inferences about the superiority of the active intervention arm (over the control). Decisions about whether to stop the study or continue recruitment, at each interim analysis w, are made by comparing the test statistic Z_w to pre-defined lower and upper boundaries l_w and u_w. These boundaries are such that the type I error rate (false positive rate) is controlled across all W interim analyses. For a one-sided alternative at overall level α, with possible stopping for futility, the type I error rate spent is such that $\alpha^*_{u1} < \cdots < \alpha^*_{uW} = \alpha$ and $\alpha^*_{l1} < \cdots < \alpha^*_{lW} = 1 - \alpha$, where α^*_{uw} is the probability of stopping and rejecting $H0$ in favour of $\beta_t > 0$ at look w (efficacy) and α^*_{lw} is the probability of stopping without rejecting $H0$ at look w (futility). The type I error rate spent is determined by α^*_{lw} and α^*_{uw}, which are specified in advance of the study beginning. For a two-arm study, standard group sequential methods and widely available software allow one to calculate the lower and upper stopping boundaries (l_w and u_w) at each look w using numerical integration [12]. For instance, the `gsBound` function in R package `gsDesign` [13], can calculate boundaries using the expected information and values of α^*_{lw} and α^*_{uw}.
As a simple illustrative example, consider a trial with two planned interim analyses ($w = 1, 2$ and total number of interim analyses $W = 2$), for two outcomes, early and late (primary) outcomes, and expected variance equal to 4 for both outcomes, and expected correlation $\rho_{12} = 0.5$ and variances $\sigma^2_1 = \sigma^2_2 = 4$. If we plan the first interim analysis when there are primary outcome data from 20 participants and early data from 40 participants in each group, then we expect that $N0_{11} = N1_{11} = 40$, $N0_{21} = N1_{21} = 20$, assuming equal numbers in intervention arms, and for notational convenience $N0_{31} = N1_{31} = 0$. Using expressions (9) to (10) and the appropriate covariance matrices, we can write $\text{var}(\beta_t) = \text{V}[t, t] = 7/20$ and the information at the first interim analysis is $I_1 = 20/7$. At the second interim analysis, we might expect that $N0_{12} = N1_{12} = 60$, $N0_{22} = N1_{22} = 30$ and $N0_{32} = N1_{32} = 0$, and thus the expected information is $I_2 = 30/7$. The information at the end of the study, which we denote as analysis $W + 1 = 3$, when $N0_{13} = N1_{13} = N0_{23} = N1_{23} = 90$ is given by $I_3 = 45/4$, or more simply in this example where sample sizes in the groups are equal (i.e. at the study end we expected that $N0_{k3} = N1_{k3} = 90$) by $I_3 = N_{k3}/2\sigma^2_2 = 45/4$. Setting the lower and upper stopping probabilities to $\alpha^*_l = (0.320, 0.640, 0.975)$ and $\alpha^*_l = (0.001, 0.010, 0.025)$ respectively, and the corresponding information as $I = (20/7, 30/7, 45/4)$, as inputs to the `gsBound` function, gives the following lower and upper bounds $l = (-0.47, 0.33, 2.06)$ and $u = (3.09, 2.34, 2.06)$. Therefore, to undertake a group-sequential study, we recruit participants and monitor current information from early and late outcomes until it first reaches I_1. We then calculate Z_1 and compare to the lower and upper boundaries l_1 and u_1, stopping the study for futility if $Z_1 \leq l_1$ or efficacy if $Z_1 \geq u_1$. Otherwise, the study continues to recruit until we reach I_2, when we stop the study for futility if $Z_2 \leq l_2$ or efficacy if $Z_2 \geq u_2$. If the study continues to the end, we reject at the upper 2.5% level if $Z_3 \geq u_3$. For studies that stop at an interim analysis, data collection will continue (until follow-up is complete) for all participants recruited up to the point the study is stopped. In such circumstances, final inferences can be made using the *deletion method* that effectively analyses the data as if the only interim analyses to have taken place were those prior to analysis w, when the study stopped, and the analysis at $w + 1$ (the overrunning analysis) [11, 14]. Bias corrected estimates and confidence intervals can be readily calculated using previously described methods [9, 10] and available R packages [15, 13, 16].

6 R code

As a means to demonstrate how the models described here can be fitted to longitudinal data from a clinical trial, code is shown below in R [7] to simulate data with $t = 3$ time-points for N individuals from two treatment groups (coded as 0, the control, and 1). Correlations are arbitrarily set, for illustrative purposes, to $\rho_{12} = 0.6$, $\rho_{23} = 0.5$

and $\rho_{13} = 0.8$ and standard deviations to $\sigma_1 = 10$, $\sigma_2 = 20$ and $\sigma_3 = 16$, and data set to missing for 50% and 25% of values at time-points $t = 3$ and $t = 2$ respectively to simulate data at an interim analysis when follow-up is incomplete. Models are fitted using functions `gls` and `lme` in package `nlme` [17]. The simulations in Section 7 use a much more general coding framework, than that shown here, that allowed different patterns of follow-up and varying treatment group sizes.

```
# R code to simulate data and fit models in order to estimate effects
# simulate data for t=3 time points
# for control (0) and intervention (1) treatment groups
# intervention effect at t=3 is 10 (at t=1 is 6 and t=2 is 4, arbitrarily)
# standard deviations at t=1,2,3 are 10,20,16
# correlations r12=0.6, r23=0.5, and r13=0.8
# load packages
library(nlme)
library(mvtnorm)
# set seed for data simulation
set.seed(78012)
set.seed(58963)
# create matrix of treatment means nrows=3 and ncols=2 (treatment groups)
mean.matrix <- matrix(c(0,6,0,4,0,10),byrow=TRUE,ncol=2)
corr.matrix <- matrix(c(1,0.6,0.8,0.6,1,0.5,0.8,0.5,1),byrow=TRUE,nrow=3)
# diagonal matrix of standard deviations
sd.matrix <- diag(c(10,20,16))
cov.matrix <- sd.matrix%*%corr.matrix%*%sd.matrix
# simulate data for N=100 participants
N <- 100
# treatment group indicator variable
treat <- rep(seq(0,1),rep(N,2))
mean.full <- matrix(rep(t(mean.matrix),each=N),byrow=FALSE,nrow=2*N)
error.full <- rmvnorm(2*N,mean=rep(0,dim(mean.matrix)[1]),
+     sigma=covariance.matrix)
# full data at study completion
full.data <- data.frame(error.full+mean.full)
# include missing values to simulate interim analysis
# 50% missing at t=3 and 25% missing at t=2 (arbitrarily)
full.data[seq(1,2*N,2),3] <- NA
full.data[seq(1,2*N,4),2] <- NA
full.data <- data.frame(id = rep(1:(2*N),each=3),
+     treat = factor(rep(rep(seq(0,1),rep(N,2)),each=3)),
+     atimes = factor(rep(1:3,times=2*N)),y=as.numeric(t(full.data[,1:3])))
# table showing data missingness
table(full.data$atimes, is.na(full.data$y)==FALSE)
# complete cases only for model fitting
complete.data <- subset(full.data, complete.cases(full.data))
# fit gls model
gls.fit <- gls(y (atimes-1)+(atimes-1):treat,data=complete.data,
+     correlation=corSymm(form =~1|id),
+     weights=varIdent(form =~1|atimes),method="REML")
summary(gls.fit)
# estimates of model coefficients and covariance matrix
# cf.  expected treatment effect at t=3 is 10
coef(gls.fit)
vcov(gls.fit)
# cf.  expected standard deviations 10,20,16
coef(gls.fit$modelStruct$varStruct,uncons=FALSE,allCoef=TRUE)*gls.fit$sigma
# cf.  expected correlations 0.6,0.8,0.5
coef(gls.fit$modelStruct$corStruct,uncons=FALSE,allCoef=TRUE)
```

```
# fit lme model
lme.fit <- lme(y (atimes-1)+(atimes-1):treat,random= 1|id,data=complete.data,
+     correlation=corSymm(form=∼1|id),
+     weights=varIdent(form=∼1|atimes),method="REML")
summary(lme.fit)
# estimates of model coefficients and covariance matrix
fixef(lme.fit)
vcov(lme.fit)
# coefficient and covariance matrix estimates are the same for gls and lme
```

7 Simulations

At an interim analysis during a clinical trial, comparing a control and test intervention, for an outcome reported at a series of t time-points, we wish to estimate the treatment effect at time β_t. In order to do this, we fit a longitudinal model (Section 2) to the totality of data available at the interim analysis. We can fit either a mixed-effects model or generalized least squares model using the functions `lme` and `gls` in R package `nlme`. We show, via a simulation study, that both methods provide identical (and consistent) estimates of β_t and $\mathrm{var}(\beta_2)$. However, function `gls` provides identifiable estimates of the covariance parameters (σ and ρ) that are useful for assessing model assumptions as a study progresses and for this reason this method may often be preferable.
Data are simulated from multivariate Normal distributions with t outcomes (where $t = 2, 3, 4$), to give a time series of t outcomes for each of N participants in a clinical trial with two intervention arms, for four different settings (see Tables 1 to 4), comprising two treatment effect sizes (mean difference in outcomes between intervention arms) and two correlation models (moderate and strong), labelled as (i) to (iv), and two sample sizes (labelled as small and large N). The sample sizes were set such that the median values for the group sizes were approximately as follows in the small N study: $N_1 = 160$ and $N_2 = 80$ for $t = 2$, $N_1 = 180$, $N_2 = 120$ and $N_3 = 60$ for $t = 3$ and $N_1 = 192$, $N_2 = 144$, $N_3 = 96$ and $N_4 = 48$ for $t = 4$. Sample sizes for the large N study were ten times larger than these values. The covariance model for each setting was determined by standard deviations (σ_s), which were (arbitrarily) selected to be an even integer in the range 10 to 30, and correlations between outcomes ($\rho_{ss'}$), which were as per Tables 1 to 4 for each setting, and (arbitrarily) set to represent either moderate or strong correlations between outcomes. The mean model for each setting was such that the mean difference between intervention arms at time t was either 0 or 10, with differences at earlier time-points taking integer values in the range -10 to 10. Again, the values are arbitrary, and were selected in order to demonstrate the consistency of estimation only. At each simulation, data were generated in R using package `mvtnorm` [15] from the set covariance model for each setting, and treatment differences β_t and variances $\mathrm{var}(\beta_t)$ were estimated using functions `gls` and `lme` in package `nlme`. In addition, for `gls` covariance parameter estimates were obtained and for `lme` the residual (within-group error), intercept and total standard deviation estimates were obtained (where $\sigma^2_{\text{total}} = \sigma^2_{\text{intercept}} + \sigma^2_{\text{residual}}$).
The results of 1000 simulations are shown in Tables 1 to 4, for each of the settings. Estimates of β_t and $\mathrm{var}(\beta_t)$ were, as expected, identical for methods `gls` and `lme`, as were estimates of σ_1 (from `gls`) and σ_{total}, which for `lme` is set by default to the standard deviation for the first time-point. The results show that treatment effect estimates β_t from the simulations were consistent with the selected mean models for all values of t, and confidence intervals were narrower for the large sample sizes, than for the small sizes. Estimates of the covariance parameters (σ and ρ), from `gls`, were also consistent with the selected covariance models for all values of t. In summary, model fitting using either `gls` or `lme` gives exactly the same overall model fit. For applications, where monitoring information accumulation and assessing modelling assumptions for the covariance parameters at interm analyses during a clinical trial, fitting models using `gls` may be preferable as it allows these tasks to be undertaken much more simply and directly.

Competing interests

All authors have previously received or are currently in receipt of funding from the National Institute of Health Research (NIHR).

Table 1: Estimates of means and confidence intervals of model parameters (based on 1000 simulations), β_2, var(β_2), $\rho_{ss'}$, σ_s and standard deviation components for settings (i) to (iv), $t = 2$ for small and large N using `gls` and `lme`

Method	Parameter	Set	Small N		Large N	
			Mean	95%CI	Mean	95%CI
(i) $\beta_2 = 0$ and moderate correlation						
`gls/lme`	β_2	0	0.104	(-0.158 - 0.365)	-0.012	(-0.095 - 0.072)
	var(β_2)	-	18.530	(18.321 - 18.740)	1.842	(1.836 - 1.848)
`gls`	ρ_{12}	0.4	0.401	(0.396 - 0.407)	0.401	(0.399 - 0.402)
	σ_1	28	27.998	(27.899 - 28.097)	28.000	(27.968 - 28.031)
	σ_2	20	19.888	(19.787 - 19.990)	19.993	(19.962 - 20.024)
`lme`	σ_{total}	28	27.998	(27.899 - 28.097)	28.000	(27.968 - 28.031)
	$\sigma_{\text{intercept}}$	-	14.727	(14.608 - 14.846)	14.655	(14.604 - 14.706)
	σ_{residual}	-	23.734	(23.634 - 23.834)	23.841	(23.802 - 23.881)
(ii) $\beta_2 = 10$ and moderate correlation						
`gls/lme`	β_2	10	9.954	(9.564 - 10.344)	10.009	(9.884 - 10.133)
	var(β_2)	-	38.917	(38.458 - 39.375)	3.851	(3.838 - 3.864)
`gls`	ρ_{12}	0.2	0.203	(0.196 - 0.210)	0.198	(0.196 - 0.200)
	σ_1	16	15.917	(15.861 - 15.973)	16.003	(15.986 - 16.020)
	σ_2	28	27.948	(27.805 - 28.090)	28.012	(27.969 - 28.055)
`lme`	σ_{total}	16	15.917	(15.861 - 15.973)	16.003	(15.986 - 16.020)
	$\sigma_{\text{intercept}}$	-	8.421	(8.277 - 8.564)	9.928	(9.793 - 10.063)
	σ_{residual}	-	13.236	(13.133 - 13.338)	12.246	(12.140 - 12.352)
(iii) $\beta_2 = 0$ and strong correlation						
`gls/lme`	β_2	0	0.350	(0.121 - 0.579)	-0.012	(-0.084 - 0.061)
	var(β_2)	-	14.567	(14.455 - 14.680)	1.440	(1.436 - 1.444)
`gls`	ρ_{12}	0.9	0.900	(0.899 - 0.901)	0.900	(0.899 - 0.900)
	σ_1	28	27.973	(27.875 - 28.070)	27.971	(27.939 - 28.003)
	σ_2	22	21.999	(21.912 - 22.086)	21.983	(21.955 - 22.011)
`lme`	σ_{total}	28	27.973	(27.875 - 28.070)	27.971	(27.939 - 28.003)
	$\sigma_{\text{intercept}}$	-	20.326	(20.232 - 20.421)	20.352	(20.316 - 20.388)
	σ_{residual}	-	19.106	(18.976 - 19.236)	19.172	(19.126 - 19.218)
(iv) $\beta_2 = 10$ and strong correlation						
`gls/lme`	β_2	10	10.008	(9.787 - 10.229)	10.033	(9.966 - 10.099)
	var(β_2)	-	12.051	(11.953 - 12.148)	1.190	(1.187 - 1.193)
`gls`	ρ_{12}	0.9	0.901	(0.899 - 0.902)	0.900	(0.900 - 0.900)
	σ_1	10	9.984	(9.950 - 10.018)	9.997	(9.986 - 10.008)
	σ_2	20	20.029	(19.949 - 20.110)	19.989	(19.964 - 20.014)
`lme`	σ_{total}	10	9.984	(9.950 - 10.018)	9.997	(9.986 - 10.008)
	$\sigma_{\text{intercept}}$	-	4.149	(4.120 - 4.179)	4.099	(4.088 - 4.109)
	σ_{residual}	-	9.064	(9.025 - 9.102)	9.116	(9.104 - 9.128)

Table 2: Estimates of means and confidence intervals of model parameters (based on 1000 simulations), β_3, var(β_3), $\rho_{ss'}$, σ_s and standard deviation components for settings (i) to (iv), $t = 3$ for small and large N using `gls` and `lme`

Method	Parameter	Set	Small N		Large N	
			Mean	95%CI	Mean	95%CI
(i) $\beta_3 = 0$ and moderate correlation						
`gls/lme`	β_3	0	0.016	(-0.265-0.297)	0.040	(-0.050-0.129)
	var(β_3)	-	19.419	(19.157-19.680)	1.926	(1.918-1.934)
`gls`	ρ_{12}	0.1	0.096	(0.090-0.102)	0.101	(0.100-0.103)
	ρ_{13}	0.4	0.393	(0.386-0.401)	0.402	(0.400-0.404)
	ρ_{23}	0.1	0.100	(0.092-0.109)	0.101	(0.099-0.104)
	σ_1	28	27.927	(27.832-28.022)	28.015	(27.986-28.043)
	σ_2	20	19.974	(19.891-20.058)	19.997	(19.973-20.022)
	σ_3	18	17.962	(17.855-18.068)	17.988	(17.956-18.020)
`lme`	σ_{total}	28	27.927	(27.832-28.022)	28.015	(27.986-28.043)
	$\sigma_{\text{intercept}}$	-	9.458	(9.385-9.531)	9.482	(9.460-9.505)
	σ_{residual}	-	26.247	(26.148-26.345)	26.358	(26.329-26.388)
(ii) $\beta_3 = 10$ and moderate correlation						
`gls/lme`	β_3	10	10.031	(9.758-10.303)	9.991	(9.906-10.076)
	var(β_3)	-	18.889	(18.645-19.133)	1.870	(1.863-1.878)
`gls`	ρ_{12}	0.5	0.499	(0.495-0.503)	0.500	(0.499-0.501)
	ρ_{13}	0.2	0.203	(0.195-0.210)	0.198	(0.196-0.201)
	ρ_{23}	0.5	0.501	(0.495-0.507)	0.499	(0.497-0.501)
	σ_1	16	16.002	(15.950-16.054)	15.994	(15.977-16.010)
	σ_2	28	27.897	(27.783-28.012)	28.022	(27.987-28.057)
	σ_3	18	17.974	(17.868-18.081)	17.992	(17.960-18.024)
`lme`	σ_{total}	16	16.002	(15.950-16.054)	15.994	(15.977-16.010)
	$\sigma_{\text{intercept}}$	-	8.108	(8.050-8.166)	7.988	(7.968-8.009)
	σ_{residual}	-	13.764	(13.712-13.817)	13.851	(13.831-13.870)
(iii) $\beta_3 = 0$ and strong correlation						
`gls/lme`	β_3	0	-0.142	(-0.434-0.149)	0.065	(-0.022-0.152)
	var(β_3)	-	20.818	(20.642-20.994)	2.073	(2.067-2.078)
`gls`	ρ_{12}	0.7	0.697	(0.694-0.700)	0.700	(0.699-0.701)
	ρ_{13}	0.9	0.901	(0.899-0.902)	0.900	(0.900-0.901)
	ρ_{23}	0.6	0.600	(0.596-0.604)	0.601	(0.599-0.602)
	σ_1	28	27.893	(27.802-27.985)	27.983	(27.953-28.013)
	σ_2	22	21.905	(21.823-21.988)	21.972	(21.945-21.999)
	σ_3	26	25.985	(25.873-26.096)	26.007	(25.970-26.044)
`lme`	σ_{total}	28	27.893	(27.802-27.985)	27.983	(27.953-28.013)
	$\sigma_{\text{intercept}}$	-	16.327	(16.211-16.443)	16.789	(16.720-16.858)
	σ_{residual}	-	22.537	(22.445-22.630)	22.352	(22.304-22.399)
(iv) $\beta_3 = 10$ and strong correlation						
`gls/lme`	β_3	10	10.009	(9.707-10.312)	10.063	(9.966-10.160)
	var(β_3)	-	24.053	(23.840-24.265)	2.396	(2.390-2.403)
`gls`	ρ_{12}	0.6	0.594	(0.590-0.597)	0.598	(0.597-0.600)
	ρ_{13}	0.9	0.899	(0.898-0.901)	0.900	(0.900-0.900)
	ρ_{23}	0.5	0.493	(0.489-0.498)	0.499	(0.498-0.500)
	σ_1	10	9.948	(9.914-9.981)	9.998	(9.988-10.008)
	σ_2	20	19.911	(19.833-19.989)	20.008	(19.984-20.032)
	σ_3	28	27.897	(27.773-28.021)	27.987	(27.950-28.025)
`lme`	σ_{total}	10	9.948	(9.914-9.981)	9.998	(9.988-10.008)
	$\sigma_{\text{intercept}}$	-	3.968	(3.943-3.994)	4.095	(4.087-4.104)
	σ_{residual}	-	9.112	(9.078-9.147)	9.119	(9.109-9.130)

Table 3: Estimates of means and confidence intervals of model parameters (based on 1000 simulations), β_4, var(β_4), $\rho_{ss'}$, σ_s and standard deviation components for settings (i) to (iii), $t = 4$ for small and large N using `gls` and `lme`

Method	Parameter	Set	Small N		Large N	
			Mean	95%CI	Mean	95%CI
(i) $\beta_4 = 0$ and moderate correlation						
`gls/lme`	β_4	0	0.091	(-0.190-0.371)	0.067	(-0.018-0.151)
	var(β_4)	-	18.544	(18.268-18.819)	1.878	(1.869-1.887)
`gls`	ρ_{12}	0.1	0.099	(0.093-0.104)	0.099	(0.097-0.100)
	ρ_{13}	0.1	0.097	(0.091-0.103)	0.099	(0.098-0.101)
	ρ_{14}	0.4	0.398	(0.390-0.405)	0.399	(0.396-0.401)
	ρ_{23}	0.3	0.293	(0.287-0.299)	0.298	(0.296-0.300)
	ρ_{24}	0.1	0.108	(0.099-0.117)	0.100	(0.098-0.103)
	ρ_{34}	0.1	0.101	(0.092-0.110)	0.098	(0.095-0.101)
	σ_1	28	28.018	(27.931-28.106)	28.004	(27.976-28.031)
	σ_2	20	19.954	(19.881-20.026)	19.994	(19.970-20.018)
	σ_3	18	17.919	(17.840-17.998)	17.998	(17.972-18.023)
	σ_4	16	15.933	(15.830-16.035)	16.017	(15.985-16.048)
`lme`	σ_{total}	28	28.018	(27.931-28.106)	28.004	(27.976-28.031)
	$\sigma_{\text{intercept}}$	-	7.562	(7.500-7.624)	7.577	(7.559-7.595)
	σ_{residual}	-	26.957	(26.867-27.048)	26.957	(26.929-26.985)
(ii) $\beta_4 = 10$ and moderate correlation						
`gls/lme`	β_4	10	9.609	(9.107-10.110)	10.085	(9.938-10.233)
	var(β_4)	-	60.359	(59.451-61.266)	6.101	(6.074-6.128)
`gls`	ρ_{12}	0.2	0.198	(0.193-0.204)	0.198	(0.196-0.200)
	ρ_{13}	0.5	0.495	(0.491-0.500)	0.500	(0.499-0.501)
	ρ_{14}	0.2	0.198	(0.190-0.206)	0.199	(0.196-0.201)
	ρ_{23}	0.5	0.502	(0.498-0.507)	0.499	(0.497-0.500)
	ρ_{24}	0.5	0.496	(0.489-0.503)	0.497	(0.495-0.499)
	ρ_{34}	0.4	0.398	(0.390-0.405)	0.400	(0.397-0.402)
	σ_1	16	15.951	(15.898-16.004)	16.001	(15.985-16.017)
	σ_2	28	28.015	(27.914-28.117)	27.970	(27.937-28.003)
	σ_3	18	17.971	(17.891-18.051)	17.983	(17.958-18.007)
	σ_4	30	29.796	(29.589-30.003)	29.960	(29.901-30.018)
`lme`	σ_{total}	16	15.951	(15.898-16.004)	16.001	(15.985-16.017)
	$\sigma_{\text{intercept}}$	-	7.261	(7.198-7.323)	7.234	(7.214-7.253)
	σ_{residual}	-	14.170	(14.120-14.220)	14.269	(14.256-14.283)
(iii) $\beta_4 = 0$ and strong correlation						
`gls/lme`	β_4	0	-0.050	(-0.213-0.112)	-0.008	(-0.059-0.042)
	var(β_4)	-	6.961	(6.890-7.032)	0.694	(0.692-0.696)
`gls`	ρ_{12}	0.8	0.799	(0.798-0.801)	0.800	(0.799-0.800)
	ρ_{13}	0.6	0.598	(0.594-0.601)	0.599	(0.598-0.600)
	ρ_{14}	0.8	0.800	(0.797-0.803)	0.800	(0.799-0.800)
	ρ_{23}	0.6	0.600	(0.596-0.603)	0.599	(0.598-0.601)
	ρ_{24}	0.5	0.500	(0.496-0.505)	0.500	(0.499-0.501)
	ρ_{34}	0.7	0.698	(0.694-0.702)	0.700	(0.699-0.702)
	σ_1	28	27.981	(27.894-28.068)	27.997	(27.969-28.025)
	σ_2	22	21.968	(21.892-22.044)	21.976	(21.952-22.001)
	σ_3	26	25.950	(25.836-26.064)	25.987	(25.950-26.023)
	σ_4	14	14.003	(13.932-14.073)	13.990	(13.970-14.010)
`lme`	σ_{total}	28	27.981	(27.894-28.068)	27.997	(27.969-28.025)
	$\sigma_{\text{intercept}}$	-	6.499	(6.443-6.555)	6.542	(6.519-6.565)
	σ_{residual}	-	27.198	(27.108-27.289)	27.219	(27.189-27.249)

Table 4: Estimates of means and confidence intervals of model parameters (based on 1000 simulations), β_4, var(β_4), $\rho_{ss'}$, σ_s and standard deviation components for setting (iv), $t = 4$ for small and large N using `gls` and `lme`

Method	Parameter	Set	Small N		Large N	
			Mean	95%CI	Mean	95%CI
(iv) $\beta_4 = 10$ and strong correlation						
`gls/lme`	β_4	10	9.936	(9.713-10.158)	9.940	(9.868-10.011)
	var(β_4)	-	12.483	(12.345-12.621)	1.250	(1.246-1.254)
`gls`	ρ_{12}	0.5	0.499	(0.495-0.503)	0.500	(0.499-0.502)
	ρ_{13}	0.5	0.501	(0.497-0.506)	0.501	(0.500-0.503)
	ρ_{14}	0.8	0.800	(0.797-0.803)	0.800	(0.800-0.801)
	ρ_{23}	0.8	0.799	(0.797-0.801)	0.800	(0.799-0.801)
	ρ_{24}	0.4	0.397	(0.392-0.403)	0.400	(0.398-0.401)
	ρ_{34}	0.6	0.599	(0.594-0.604)	0.600	(0.598-0.601)
	σ_1	10	10.003	(9.971-10.035)	10.000	(9.989-10.010)
	σ_2	20	19.972	(19.899-20.046)	20.006	(19.982-20.029)
	σ_3	28	28.004	(27.886-28.122)	28.008	(27.970-28.046)
	σ_4	18	17.986	(17.892-18.081)	18.016	(17.987-18.045)
`lme`	σ_{total}	10	10.003	(9.971-10.035)	10.000	(9.989-10.010)
	$\sigma_{\text{intercept}}$	-	5.091	(5.053-5.128)	5.179	(5.166-5.192)
	σ_{residual}	-	8.594	(8.568-8.621)	8.551	(8.542-8.561)

References

[1] Parsons, N., Stallard, N., Parsons, H., Wells, P., Underwood, M., Mason, J., Metcalfe, A.: An adaptive two-arm clinical trial using early endpoints to inform decision making: design for a study of sub-acromial spacers for repair of rotator cuff tendon tears. Trials **20**(1), 694 (2019). doi:10.1186/s13063-019-3708-6

[2] Engel, B., Walstra, P.: Increasing precision or reducing expense in regression experiments by using information from a concomitant variable. Biometrics **47**(1), 13–20 (1991). doi:10.2307/2532491

[3] Galbraith, S., Marschner, I.C.: Interim analysis of continuous long-term endpoints in clinical trials with longitudinal outcomes. Statistics in Medicine **22 11**, 1787–805 (2003)

[4] Spiessens, B., Lesaffre, E., Verbeke, G., Kim, K., DeMets, D.L.: An overview of group sequential methods in longitudinal clinical trials. Statistical Methods in Medical Research **9**(5), 497–515 (2000)

[5] Spiessens, B., Lesaffre, E., Verbeke, G.: A comparison of group sequential methods for binary longitudinal data. Statistics in Medicine **22**(4), 501–515 (2003)

[6] Diggle, P., Diggle, P.: Analysis of Longitudinal Data, 2nd edn. Oxford statistical science series, p. 379. Oxford University Press, Oxford ; New York (2002)

[7] R Core Team: R: A Language and Environment for Statistical Computing. R Foundation for Statistical Computing, Vienna, Austria (2022). R Foundation for Statistical Computing. https://www.R-project.org/

[8] Pinheiro, J.C., Bates, D.: Mixed-Effects Models in S and S-PLUS. Statistics and Computing, p. 528. Springer, New York (2009)

[9] Todd, S., Whitehead, J., Facey, K.M.: Point and interval estimation following a sequential clinical trial. Biometrika **83**(2), 453–461 (1996)

[10] Liu, A., Hall, W.: Unbiased estimation following a group sequential test. Biometrika **86**(1), 71–78 (1999)

[11] Whitehead, J.: Overrunning and underrunning in sequential clinical trials. Controlled Clinical Trials **13 2**, 106–21 (1992)

[12] Jennison, C., Turnbull, B.W.: Group Sequential Methods with Applications to Clinical Trials, p. 390. Chapman and Hall, Boca Raton (2000)

[13] Anderson, K.: gsDesign: Group Sequential Design (https://CRAN.R-project.org/package=gsDesign). (2021). https://CRAN.R-project.org/package=gsDesign

[14] Sooriyarachchi, M.R., Whitehead, J., , Bolland, K., Whitehead, A.: Incorporating data received after a sequential trial has stopped into the final analysis: Implementation and comparison of methods. Biometrics **59**(3), 701–709 (2003)

[15] Genz, A., Bretz, F., Miwa, T., Mi, X., Leisch, F., Scheipl, F., Hothorn, T.: mvtnorm: Multivariate Normal and T Distributions (http://CRAN.R-project.org/package=mvtnorm). (2021). http://CRAN.R-project.org/package=mvtnorm

[16] Galarza, C.E., Kan, R., Lachos, V.H.: MomTrunc: Moments of Folded and Doubly Truncated Multivariate Distributions. (2021). R package version 5.97. https://CRAN.R-project.org/package=MomTrunc

[17] Pinheiro, J., Bates, D., DebRoy, S., Sarkar, D., Core Team, R.: nlme: Linear and Nonlinear Mixed Effects Models (https://CRAN.R-project.org/package=nlme). (2021). https://CRAN.R-project.org/package=nlme
